# Investigation of certain miRNA expression levels in bovine mastitis cases caused by *Escherichia coli* and Coagulase-Negative Staphylococci

**DOI:** 10.1371/journal.pone.0352609

**Published:** 2026-07-29

**Authors:** Behnoush Khasheii, Pezhman Mahmoodi, Taghi Zahraei Salehi, Abdolmajid Mohammadzadeh, Ali Sadeghi-nasab, Morteza Yavari

**Affiliations:** 1 Department of Pathobiology, Faculty of Veterinary Medicine, Bu-Ali Sina University, Hamedan, Iran; 2 Department of Microbiology and Immunology, Faculty of Veterinary Medicine, University of Tehran, Tehran, Iran; 3 Department of Clinical Sciences, Faculty of Veterinary Medicine, Bu-Ali Sina University, Hamedan, Iran; Cornell University, UNITED STATES OF AMERICA

## Abstract

MicroRNAs (miRNAs) are small, non-coding RNA molecules approximately 18–25 nucleotides in length that function as essential regulators in numerous biological processes, including immune system function and inflammatory conditions such as bovine mastitis. They hold considerable potential as biomarkers for this disease. This study aimed to investigate the expression levels of inflammation-associated miRNAs in the milk of cows with clinical and subclinical mastitis. In this study, 15 whole milk samples were collected from dairy cows, comprising three groups: clinical mastitis (N = 5), subclinical mastitis (N = 5), and healthy (N = 5). The expression levels of nine inflammation-related miRNAs (miR-146a, miR-92a, miR-155, miR-383, miR-29B-2, miR-223, miR-148a, miR-200a, and miR-205) were measured and analyzed using real-time quantitative PCR (qPCR). The results revealed a significant upregulation in the expression levels of miR-92a, miR-155, miR-223, and miR-200a in clinical mastitis cases induced by *Escherichia coli* infection. In contrast, subclinical mastitis caused by coagulase-negativestaphylococci (CoNS) showed a significant downregulation of miR-146a, miR-92a, miR-148a, and miR-205. Furthermore, the expression levels of miR-223 and miR-200a were significantly upregulated in both clinical and subclinical forms of the disease. The findings of this study revealed significant alterations in the expression levels of miR-223 and miR-200a, particularly in clinical mastitis, which highlights greater discriminatory power of these miRNAs as diagnostic biomarkers. Accordingly, milk miRNAs appear to have considerable potential for use as non-invasive biomarkers in the early and differential diagnosis of mastitis.

## Introduction

Mastitis is an infectious disease of the mammary glands characterized by inflammation of the udder tissue in dairy cattle, leading to reduced milk yield and quality [1]. It causes significant economic losses in the global dairy industry [2]. Controlling mastitis remains a global challenge that complicates animal health management [3]. Bovine mastitis is an inflammatory disease classified into clinical and subclinical types based on its characteristics [4]. The etiological agents responsible for the disease may include bacteria, viruses, or fungi, among others. [5]. Milk production often drops as a direct consequence of either the pathogen itself or damage inflicted by the immune response. [6]. The principal bacterial pathogens include *Staphylococcus* (Gram-positive) and *Escherichia coli* (Gram-negative) [7]. Mastitis induced by *E. coli* and other Gram-negative bacteria typically manifests as a clinical condition accompanied by acute and severe inflammation of the mammary gland. The pathogens are subsequently cleared by the host immune system within a few days or following antibiotic therapy [8]. In contrast, mastitis caused by Gram-positive bacteria such as *Staphylococcus aureus* is often milder; however, antibiotic treatment is frequently ineffective [9], leading to chronic clinical mastitis [10]. Coagulase-negative staphylococci: CoNS (e.g., *S. simulans*, *S. saprophyticus*, *S. chromogenes*, *S. epidermidis*) are opportunistic pathogens that colonize the teat canal lining and teat skin. Staphylococcal mastitis is one of the most prevalent causes of economic losses due to its impact on reducing milk production [11].

Understanding the mechanisms of the host response to these infectious pathogens and developing appropriate control strategies can play vital roles in mastitis control. The host response to infection has demonstrated significant differences between Gram-negative (*E. coli*) and Gram-positive (staphylococci) bacteria [12]. Furthermore, these pathogens have been shown to elicit markedly distinct mechanisms within the host innate immune response [13]. Following infection with *E. coli* or *S. aureus*, bovine mammary epithelial cells and tissues mount an immune response characterized by the secretion of various cytokines, chemokines, and other specific proteins, which are also detectable in milk [14,15].

The conventional gold-standard methods for diagnosing mastitis including microbial culture, somatic cell count (SCC), and California Mastitis Test (CMT) remain in widespread use. Although these techniques are inexpensive, rapid, and simple to perform, they are consistently associated with limitations in terms of diagnostic accuracy and reliability [16,17]. Currently, mastitis diagnostic methodologies are undergoing modern advancements. Recently, regulatory biomarkers, such as MicroRNAs (miRNAs), have attracted considerable scientific interest [18].

MicroRNAs are endogenous, short non-coding regulatory RNAs (approximately 18–25 nucleotides in length) that regulate gene expression at the post-transcriptional level by binding to fully or partially complementary sites within the 3'UTR of target mRNAs [19]. MicroRNAs modulate the expression of host immune genes which in turn, can substantialy influence host immunity response during mastitis, and a comprehensive understanding of their activity may help in elucidating the etiology of this disease [20].

Although miRNAs are crucial regulators of the host's response to infectious agents [21], few studies have investigated how bovine miRNA expression levels alter in response to such infections [22]. Currently, efforts have been made to utilize diagnostic biomarkers such as miRNAs, which are present in the microvesicles of milk from dairy cattle. The identification of these biomarkers could provide a more effective alternative method for diagnosis of mastitis [23]. Furthermore, milk contains the highest abundance of miRNAs among various bodily fluids [24]. Studies on miRNA expression changes during bacterial mastitis have demonstrated that stimulating bovine CD14^+^ monocytes with lipopolysaccharide (LPS) or *Staphylococcus aureus* enterotoxin B (SEB) leads to differential expression of five key inflammation-related miRNAs: miR-9, miR-125b, miR-155, miR-146a, and miR-223 [25]. Moreover, another study reported altered expression of five miRNAs (bta-miR-184, miR-24-3p, miR-148, miR-486, and let-7a-5p) in response to experimental intramammary infection with *E. coli* [26]. Similarly, in an investigation of mastitis induced by *Streptococcus uberis*, four out of fourteen studied miRNAs (miR-181a, miR-16, miR-31, and miR-223), which are associated with innate immune regulation and mammary gland function, were found to be differentially regulated [27]. Despite this advantage, the application of milk miRNA expression profiling for the diagnosis of bovine mastitis remains relatively unexplored, with only a limited number of studies addressing this potential [28,29]. The KEGG pathways significantly enriched for differentially expressed miRNA targets are established mediators of the bovine mammary gland's response to bacterial pathogens [22,27]. As an example, a key regulator of innate immunity, miR-146a attenuates the response to bacterial infection by suppressing the expression of TRAF6 and IRAK1 [30]. This molecule acts as a negative feedback regulator by modulating the TLR4/TRAF6/NF-κB signaling pathway during inflammation [31].Current techniques for miRNA detection include conventional methods such as northern blotting [32], microarray [33], and qPCR [34], as well as newer approaches such as digital PCR and small RNA sequencing [35,36]. Nevertheless, the application of miRNAs as biomarkers for the diagnosis and confirmation of bovine mastitis directly from milk has not been extensively studied [37]. The potential of miRNAs in diagnosing or predicting livestock diseases remains incompletely understood [20,38]. Furthermore, investigations into the alterations in miRNA expression profiles in milk from healthy cows and those with naturally acquired subclinical and clinical mastitis remain scarce. Therefore, advancing bovine mastitis research by integrating novel biomarkers with conventional methods may present a promising strategy for enhancing diagnostic and therapeutic outcomes. Accordingly, we selected certain miRNAs that have been most frequently reported in the literature to be associated with inflammatory responses in bovine mastitis. The aim of this study was to employ qPCR to analyze the expression dynamics of these miRNAs in milk samples from naturally infected and healthy cows, comparing three distinct groups: clinical mastitis caused by *E. coli*, subclinical mastitis associated with coagulase negative staphylococci, and apparently healthy controls.

## Materials and methods

### Ethical approval

The study's animal experiments adhered to the ethical protocols approved by the Animal Research Ethics Committee of Bu-Ali Sina University's Faculty of Veterinary Medicine, Hamadan, Iran. (Approval Code: IR.BASU.REC.1402.035).

### Animals

This study was conducted on at least 100 Holstein-Friesian cows (average age 3.5 years, body weight 600–750 kg).. The animals were between 60 and 120 days in milk (DIM), with daily milk yields ranging from 36 to 45 kg. Mean parity ranged from 1 to 3. All cows were apparently healthy at the time of sampling and had no history of lameness or metritis during their most recent health check.The animals were housed in free-stall barns at commercial dairy farms. All cows had ad libitum access to feed and water to satisfy their daily nutritional requirements and were milked three times daily.

### Collection of milk samples

To collect milk samples; at first, the tip of the teats was disinfected three times in a row. Then, pre-milking was performed and the initial pre-milking streams were discarded. After that, at least 20 mL of mid-stream milk was collected from the quarters using the jet milking method.

**Fig 1:**
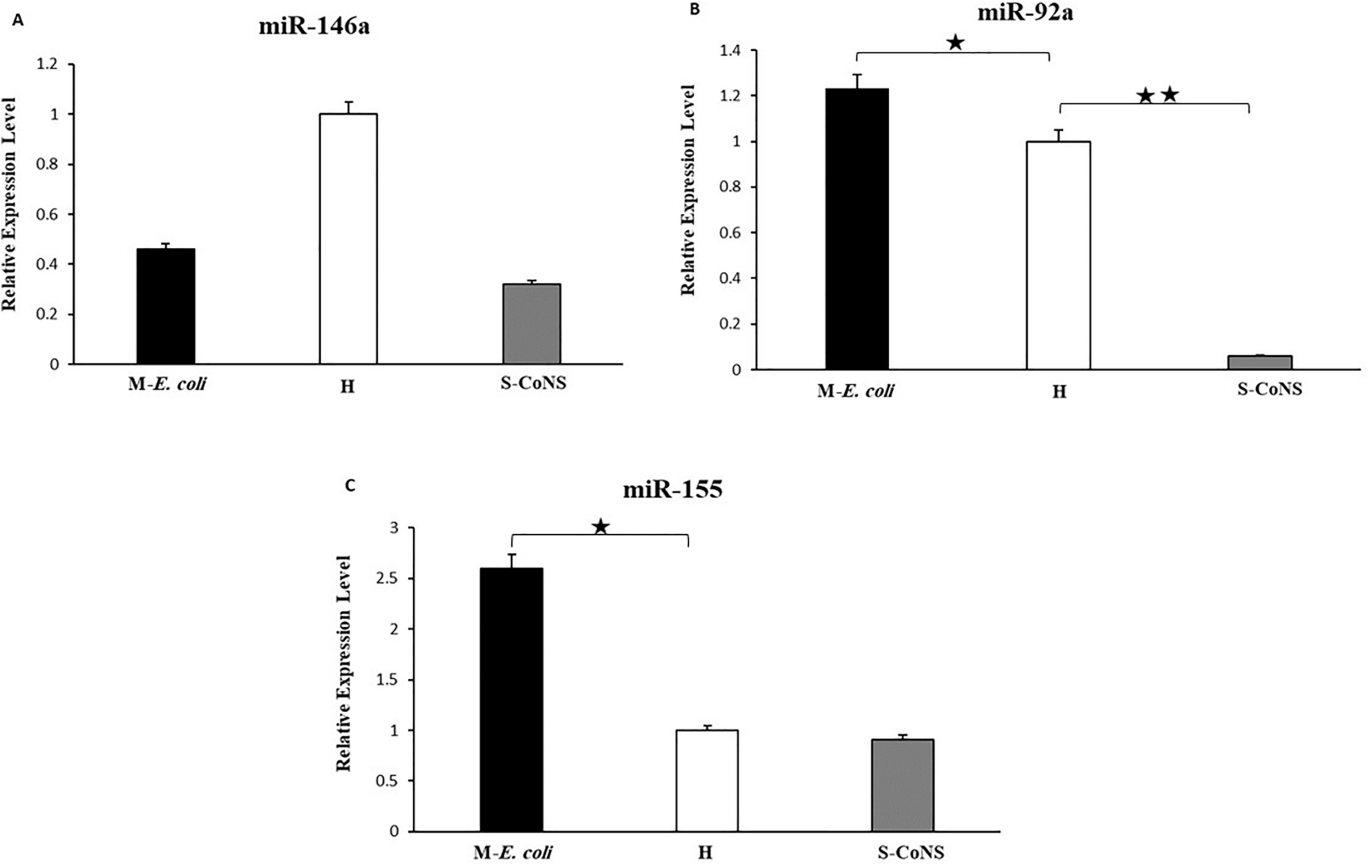
Relative expression profiles of miR-146a, miR-92a, and miR-155 in milk samples from healthy (H), clinical mastitis (M-*E. coli*), and subclinical mastitis (S-CoNS) groups. Data are presented as mean ± SE and were compared using the unpaired Student's *t*-test and Mann-Whitney U test, * p < 0.05, ** p < 0.01, *** p < 0.001, **** p < 0.0001.

**Fig 2:**
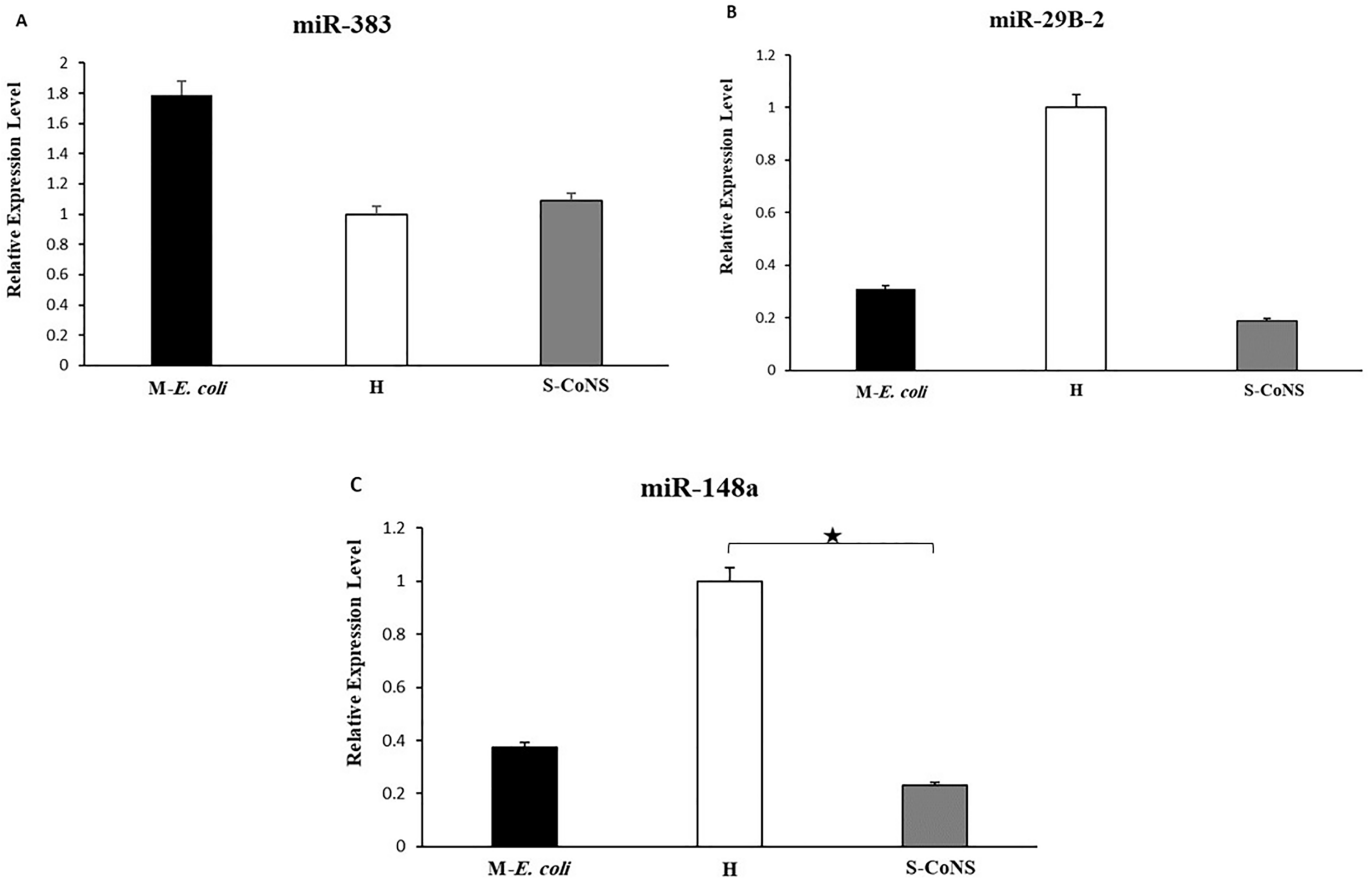
Relative expression profiles of miR-383, miR-29B-2, and miR-148a in milk samples from healthy (H), clinical mastitis (M-*E. coli*), and subclinical mastitis (S-CoNS) groups. Data are presented as mean ± SE and were compared using the unpaired Student's *t*-test and Mann-Whitney U test, * p < 0.05, ** p < 0.01, *** p < 0.001, **** p < 0.0001.

**Fig 3:**
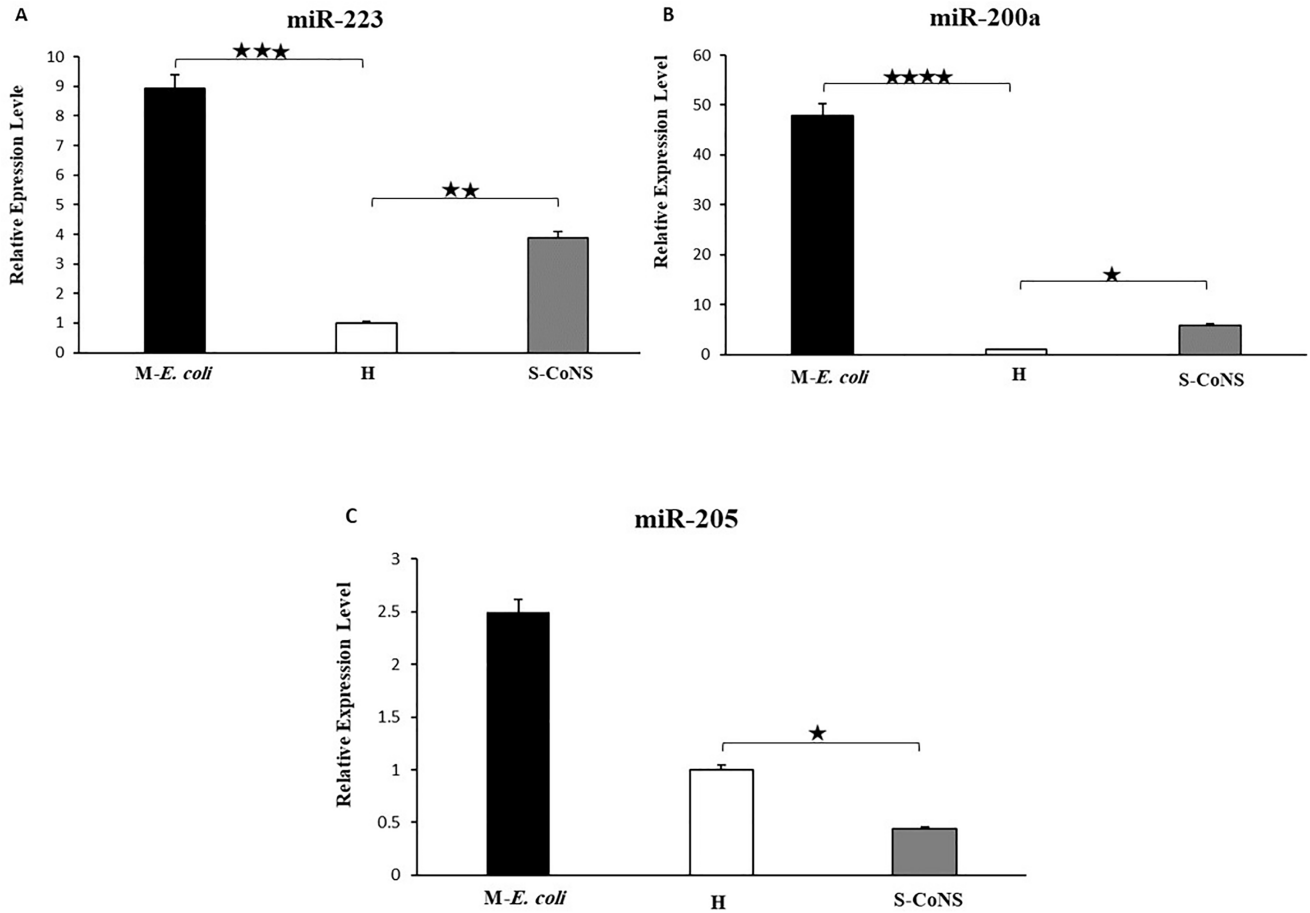
Relative expression profiles of miR-223, miR-200a, and miR-205 in milk samples from healthy (H), clinical mastitis (M-*E. coli*), and subclinical mastitis (S-CoNS) groups. Data are presented as mean ± SE and were compared using the unpaired Student's *t*-test and Mann-Whitney *U* test, * p < 0.05, ** p < 0.01, *** p < 0.001, **** p < 0.0001.

Whole milk samples were collected from at least 100 cows (one sample per cow) between January 2024 and February 2025 to obtain the final 15 milk samples (three groups including: clinical mastitis, subclinical mastitis, and apparently healthy) with certain criteria. All cows were examined for clinical signs of mastitis and all milk samples were screened for pathogenic bacteria and subjected to the California mastitis test (CMT) and somatic cell count (SCC) according to the methods described by Bergonier et al [39,40]. For the final analysis, a subset of 15 *Mycoplasma*-free (tested by a direct PCR assay: S1) milk samples was selected as follows: 1) Clinical Mastitis: five milk samples from cows exhibiting clinical signs such as redness, pain upon palpation, swelling, and flakes in the milk, with positive CMT results, high SCC, and confirmed pure *E. coli* culture, were designated as the clinical mastitis group. 2) Subclinical mastitis: five milk samples from cows without clinical symptoms but with a CMT score of ++ and SCC ranging from 200,000–500,000 cells/mL were classified as the subclinical mastitis group. These milk samples were positive for coagulase-negative staphylococci (CoNS) in pure bacterial culture. 3) Apparently healthy: five milk samples from apparently healthy cows without any clinical signs, and with negative bacterial culture, negative CMT result, and SCC below 200,000 cells/mL, were assigned as the control group (S1 Fig 1, 2 and 3 in S1 File).

### Identification of bacterial pathogens and PCR

For the primary identification of *E. coli* and coagulase-negative staphylococci (CoNS), milk samples were examined for phenotypic characteristics, including colony morphology, Gram staining, and biochemical tests. Initially, 100 µL of each milk sample was cultured on blood agar, MacConkeyagar, and mannitol salt agar plates, which were then aerobically incubated at 37°C for 48 hours. Following the incubation period, the colonies underwent further identification steps. Ultimately, the results obtained from the culture methods were confirmed by PCR using specific bacterial genes: the *uspA* gene for *E. coli* [41], and the *tuf* gene for coagulase-negative staphylococci [42], It should be noted that all of the studied milk samples were also screened for *Mycoplasma* infection using direct DNA extraction followed by a *Mycoplasma* genus-specific PCR assay [43], and consequently, all of the included samples were free of *Mycoplasma* (S1 Tables 1 and 2 and 3 in S1 File).

### RNA extraction and cDNA Synthesis

Total RNA was extracted directly from unprocessed whole milk (raw milk)samples without any prior manipulation (e.g., no centrifugation, fat removal, cell depletion, or exosome isolation) [44], using the RNX-plus kit (SinaClon, Iran) according to the manufacturer's protocol.. The concentration and purity of the extracted RNAs were measured with a NanoDrop 2000 spectrophotometer (Thermo Fisher Scientific, USA) by assessing the absorbance ratio at 260/280 nm. cDNA synthesis was performed separately for each miRNA using specific stem-loop primers and the Yekta Tajhiz kit, following the manufacturer's instructions.

### Bioinformatic primer design

The primer design was done based on data deposited in MiRBase release 21, a searchable database of published miRNA sequences, and annotation of bovine miRNAs (http://www.mirbase.org; accessed on 27 October 2025). The primers were designed from the stem-loop sequence of the corresponding miRNA by placing the forward and reverse primer flanking to the mature miRNA. The primers for inflammatory miRNA genes (bta-miR-146a, miR-29B-2, miR-148a, miR-223, miR-155, miR-383, miR-200a, miR-205, miR-92a) [25] were designed using the following web server (http://www.srnaprimerdb.com/; accessed on 27 October 2025) and the bovine S18 rRNA was used as a miRNA control [45,46]. Real-time PCR primers were synthesized by TAG Copenhagen, Denmark. The sequences of oligonucleotide primers used in this study are outlined in Table 1.

**Table 1:** Sequences of the primers designed in this study.

Primer Sequences(5’–3’)		miRNA
F: 5^׳^ - AACACGCTGTCAGTTTGTCAA −3^׳^		**miR-223**
GTCGTATCCAGTGCAGGGTCCGAGGTATTCGCACTGGATACGACTGGGGT	Stem-loop	
F: 5^׳^- AACACGCTCCTTCATTCCAC −3^׳^		**miR-205**
GTCGTATCCAGTGCAGGGTCCGAGGTATTCGCACTGGATACGACCAGACT	Stem-loop	
F: 5^׳^ - AAGCGCCTTTAATGCTAATCGT −3^׳^		**miR-155**
GTCGTATCCAGTGCAGGGTCCGAGGTATTCGCACTGGATACGACACCCCT	Stem-loop	
F: 5^׳^ - AACACGCTGAGAACTGAATTCC −3^׳^		**miR-146a**
GTCGTATCCAGTGCAGGGTCCGAGGTATTCGCACTGGATACGACACAACC	Stem-loop	
F: 5^׳^ - AACACGCTAACACTGTCTGGTA −3^׳^		**miR-200a**
GTCGTATCCAGTGCAGGGTCCGAGGTATTCGCACTGGATACGACAACATC	Stem-loop	
F: 5^׳^ - AACACGCAGATCAGAAGGTGA −3^׳^		**miR-383**
GTCGTATCCAGTGCAGGGTCCGAGGTATTCGCACTGGATACGACAGCCAC	Stem-loop	
F: 5^׳^ - AACACGCTCAGTGCACTACA −3^׳^		**miR-148a**
GTCGTATCCAGTGCAGGGTCCGAGGTATTCGCACTGGATACGACACAAAG	Stem-loop	
F: 5^׳^ - AACACGCTAGCACCATTTGAAA −3^׳^		**miR-29B-2**
GTCGTATCCAGTGCAGGGTCCGAGGTATTCGCACTGGATACGACAACACT	Stem-loop	
F: 5׳ -AACACGTGTATTGCACTTGTCC-3׳		**miR-92a**
GTCGTATCCAGTGCAGGGTCCGAGGTATTCGCACTGGATACGACACAGGC	Stem-loop	
R: 5׳- GTCGTATCCAGTGCAGGGT −3׳		**Universal reverse**
F: 5^׳^ -CACCGAGGATGAGGTGGA-3^׳^	**S18 rRNA**	**Internal control**
R: 5^׳^-TATTGGCGTGGATTCTGC-3^׳^		

**Reverse Transcription Quantitative PCR (RT-qPCR)** The expression levels of miR-146a, miR-92a, miR-155, miR-29B-2, miR-223, miR-148a, miR-205, miR-200a, and miR-383 were quantified using a StepOnePlus Real-Time PCR System (ThermoFisher Scientific, USA) and a SYBR Green PCR kit (Yekta Tajhiz, Iran). In this study, a total of nine miRNAs, including miR-146a, miR-92a, miR-155, miR-383, miR-29b-2, miR-223, miR-148a, miR-200a, and miR-205, were selected based on their importance in previous studies and their important roles in response to bacterial infections, inflammation, bovine mastitis, modulation of immune responses, and regulation of innate immune responses to infection through the production and release of pro-inflammatory cytokines [30,47–50].

Real-Time PCR reactions were performed in a 20 μL reaction volume containing the following components: 10 μL of 2 × SYBR Green qPCR Mix, 0.4 μL of 50 × Passive Reference Dye, 1 μL of specific forward primer, 1 μL of universal reverse primer, and 2.5 μL of cDNA template. The final volume was adjusted to 20 μL with RNase-free ddH₂O. The thermal cycling protocol consisted of an initial denaturation at 95 °C for 5 min, followed by 35 cycles of denaturation at 94 °C for 30 s, annealing at 62 °C for 30 s, and extension at 72 °C for 30 s. Reaction specificity was confirmed by dissociation curve analysis (melting curve). All reactions were performed in triplicate. Relative gene expression was calculated based on the CT values of the inflammatory miRNA genes and S18 rRNA. The expression levels, represented as fold changes, were reported using the 2^―ΔΔCT^ method [51].

### Target genes prediction and analysis of associated pathways and regulatory networks

The putative target genes for the known, differentially expressed miRNAs identified in our study were predicted using the miRWalk version 3 server (http://mirwalk.uni-hd.de/; accessed on 29 November 2025). To refine the initial predictions and minimize false positives, we applied a rigorous filtering strategy based on three key criteria: (1) presence of a binding site within the 3’ UTR region, (2) minimum free energy (MFE < −20 kcal/mol) score, and (3) binding p-value = 1. This database allows for the prediction of potential miRNA-binding sites within the complete sequences of all known genes from four genomes: human, cattle, mouse, rat, dog, and fish [52].

To elucidate the functional pathways associated with the target genes, Kyoto Encyclopedia of Genes and Genomes (KEGG) annotation was carried out via the Database for Annotation, Visualization and Integrated Discovery (DAVID version 6.8 https://davidbioinformatics.nih.gov/; accessed on 29 November 2025) [53]. A regulatory interaction network was constructed in Cytoscape (version 3.10.4), incorporating all predicted targets of the nine miRNAs.

### Statistical analysis

All statistical analyses were performed with GraphPad Prism software (v10; San Diego, CA). Data normality was assessed using the Shapiro-Wilk test. Based on this assessment, comparisons between groups were made using either an unpaired two-tailed Student's t-test (parametric data) or the Mann-Whitney U test (non-parametric data). Results with a p-value below 0.05 were deemed statistically significant.

## Results

### Quantitative expression profiling of miRNA biomarker candidates

As described in the statistical analysis section, group comparisons were performed using the unpaired *t*-test or Mann-Whitney *U* test based on normality assessment. Our findings indicated a significant increase in the expression levels of miR-92a, miR-155, miR-223, and miR-200a in the clinical mastitis group in response to *E. coli* infection compared to the healthy controls (p < 0.05) with the expression fold change values ranged from 1.23, 2.6, 8.93, and 47.84 (S1 Table 4 in S1 File).

Significant upregulation of miR-223 and miR-200a was observed in both clinical and subclinical mastitis groups compared to healthy controls (p < 0.001). The fold change values in the subclinical mastitis group were 3.89 for miR-223 and 5.81 for miR-200a. miR-200a exhibited the most substantial upregulation among all miRNAs in both disease states.

In clinical mastitis with *E. coli* infection, a non-significant rise in expression was noted for miR-383 and miR-205 relative to the healthy group, with respective fold changes of 1.8 and 2.5 (p > 0.05). Conversely, in the subclinical mastitis group with CoNS, the observed increase in miR-383 expression (fold change = 1.08) did not reach statistical significance (p > 0.05). Although the expression levels of miR-146a, miR-29B-2, and miR-148 showed a decrease in the clinical mastitis group relative to the healthy controls, with fold changes of 0.46, 0.31, and 0.40, respectively, the differences were not statistically significant (p > 0.05) (S1 Table 5 in S1 File).

**Fig 4:**
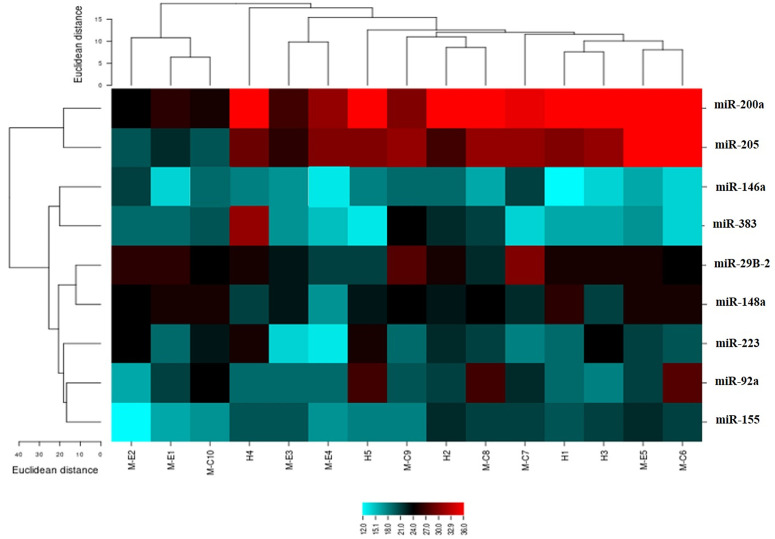
Heatmap of miRNA expression profiles generated by CIMMiner software (Euclidean distance metric). Red color intensity corresponds to the level of miRNA upregulation. Groups: M-E (Clinical mastitis, E. coli), M-C (Subclinical mastitis, CoNS), H (Healthy).

In the subclinical mastitis group infected with CoNS, a significant decrease in expression was detected for miR-92a, miR-148a, and miR-205, with respective fold changes of 0.06, 0.23, and 0.43 (p < 0.05). Meanwhile, the expression of miR-146a, miR-29B-2, and miR-155 also exhibited a declining trend, with fold changes of 0.32, 0.90, and 0.19, although these changes did not reach statistical significance (p > 0.05), (Fig 1, Fig 2, Fig 3, Fig 4).

### KEGG pathway enrichment analysis of predicted targets for DE miRNAs

**Fig 5:**
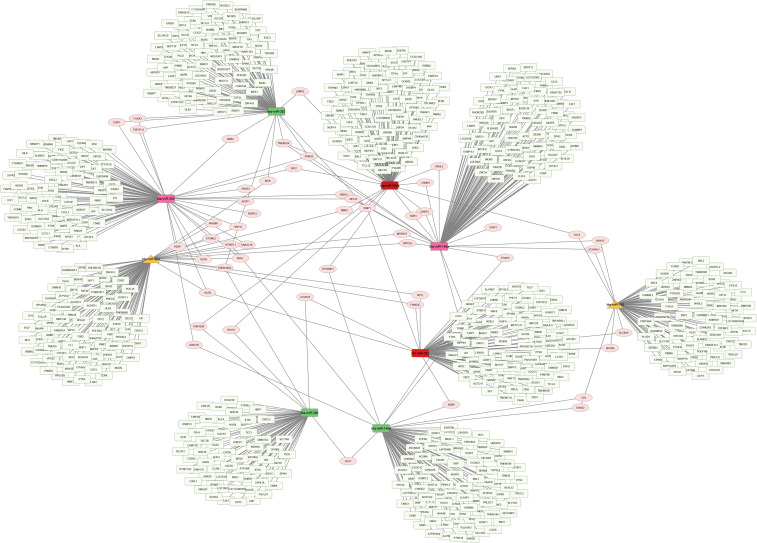
Network visualization of the regulatory interactions between differentially expressed miRNAs and their target genes in *E. coli*-induced clinical mastitis and CoNS-induced subclinical mastitis. In this representation, differentially expressed miRNAs (depicted as red, green, and pink squares and orange triangles) are connected to their respective target genes (white squares). Pink ovals highlight genes that are common targets of two or more miRNAs.

**Fig 6:**
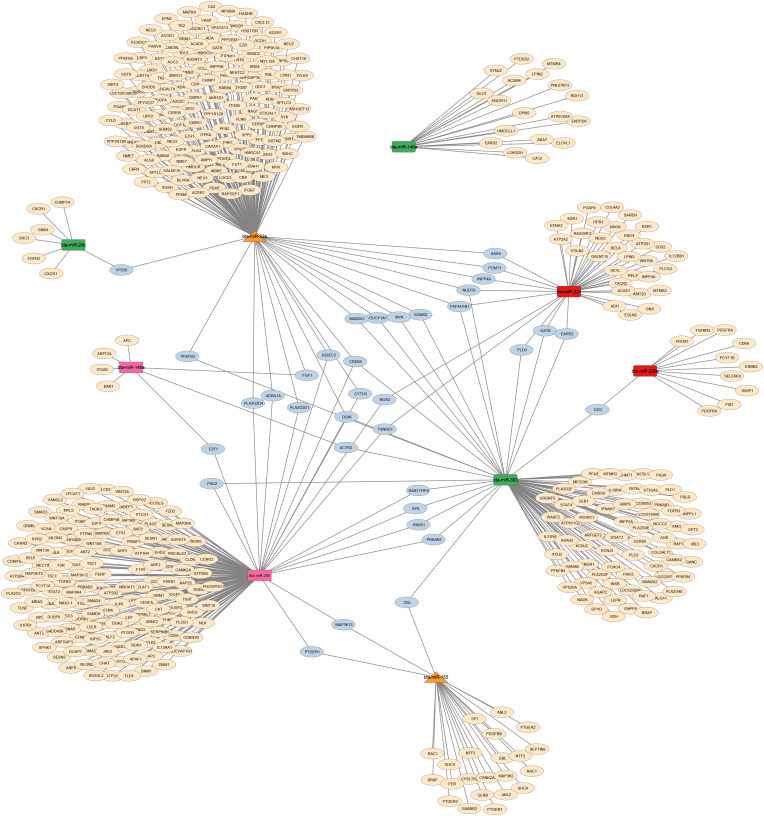
A network of differentially expressed miRNAs, along with a subset of target genes involved in pivotal pathways such as immunity, metabolic, cancer, inflammation, and bacterial invasion. In this representation, differentially expressed miRNAs (depicted as red, green, and pink squares and orange triangles) are connected to their respective target genes (yellow ovals). Blue ovals highlight genes that are common targets of two or more miRNAs.

The most significant pathways in which these miRNAs are involved include: Metabolic pathways, *mTOR* signaling pathway, *ErbB* signaling pathway, Bacterial invasion of epithelial cells, *Ras* signaling pathway, *MAPK* signaling pathway, Adherens junction, pathways in cancer, *JAK-STAT* signaling pathway, Ras signaling pathway, *AMPK* signaling pathway, Endocytosis, Chemokine signaling pathway, *HIF-1* signaling pathway, Calcium signaling pathway, Regulation of actin cytoskeleton, *p53* signaling pathway, *FoxO* signaling pathway, *Wnt* signaling pathway, *AMPK* signaling pathway, Cell adhesion moleculesm Platelet activation, and Nucleotide metabolism. Based on the analyses, it can be inferred that miRNAs such as miR-146a, miR-155, miR-92a, miR-29B-2, miR-223, miR-383, miR-148a, miR-200a, and miR-205 play significant roles in numerous crucial biological pathways. The most important of these pathways are involved in inflammatory processes and immune responses, wherein these miRNAs participate by regulating gene expression [54]) S1 Table 6 in S1 File, Fig 5 and Fig 6).

## Discussion

Evidence from the literature reveals a pattern of miRNA dysregulation in bovine mastitis. An analysis of blood by Chen et al. revealed a significant decrease in the expression of miR-148a, miR-146a, and miR-155, and an increase in the expression of miR-223 and miR-29b in mastitis cows [55]. Corroborating these findings in milk, studies by Srikok et al. and Ngo et al. confirmed the downregulation of miR-146a, miR-155, miR-148a, and miR-29b in clinical and subclinical mastitis, with miR-29b exhibiting the most pronounced decrease [37,56]. In a different model, Chuammitri et al. reported that miR-146a expression was suppressed in *E. coli* LPS-stimulated bovine neutrophils treated with quercetin [57]. In the study by Dilda et al., the expression of inflammation-related miRNAs, such as miR-155, miR-146a, and miR-223, was upregulated in bovine monocytes stimulated with *E. coli* lipopolysaccharide [25]. Similarly, Wang et al. found that the expression of miR-146a and miR-146b was increased in the mammary gland tissue of cows with clinical and subclinical mastitis [29]. Furthermore, Lai et al. reported that the expression of miR-146a, miR-383, and miR-155 was upregulated in the milk of cattle with clinical mastitis [38]. Our findings on miRNA expression are consistent with previous studies by Chen, Chuammitri, Srikok, and Ngo [37,55,57]. In our study, the expression levels of miR-146a, miR-148a, and miR-29B-2 were reduced in the milk of cows with clinical mastitis naturally infected with *E. coli* and in subclinical mastitis caused by CoNS. Among these, the downregulation of miR-148a in subclinical mastitis was significant. However, the decreases in miR-146a and miR-29B-2 expression were not significant in either clinical or subclinical mastitis. The expression pattern of miR-146a in the present study contradicted those previously reported by Lai, Wang, and Dilda. Furthermore, Srikok et al. found the downregulation of miR-29B-2 to be significant and proposed it as a potential biomarker candidate [37]. Lawless et al. also reported that the expression of miR-29B-2 was downregulated in mammary epithelial cells infected with *Streptococcus uberis* [22]. The miR-29 family plays a role in the epigenetic regulation of lactation-related genes in bovine mammary epithelial cells [48]. miR-29B acts by inhibiting the *NF-κB* pathway and *TNFAIP3* (a negative regulator of the NF-κB pathway) [49]. In our bioinformatic analysis, *CXCR1* was identified as a predicted target of miR-29B-2. This gene plays a pivotal role in the chemokine signaling pathway, suggesting a potential mechanism for immune regulation in mastitis.

In contrast to the studies by Chen and Srikok, and consistent with the findings of Dilda and, the expression level of miR-155 in our study was upregulated in clinical mastitis caused by *E. coli*, but downregulated in subclinical mastitis. This suggests that miR-155 expression in Gram-negative bacterial infections may differ from that observed in Gram-positive infections. As previously established, bovine mastitis caused by Gram-positive and -negative bacterial pathogens triggers distinct host response patterns, which consequently lead to varying degrees of mastitis severity [26]. Different miRNA response patterns to Gram-positive and -negative bacteria have also been demonstrated in previous studies [25,50]. Although there has been limited overlap among the miRNAs identified across studies, changes in the expression of a large number of miRNAs have been reported [58]. miR-155 acts as a pro-inflammatory agent, and its early upregulation during innate immunity may amplify inflammatory signaling, a response that correlates with acute inflammation following *E. coli* infection [59]. Furthermore, bioinformatics analysis demonstrated that miR-155 targets key genes, including *CBL* and *RAC1*, which are involved in important biological pathways such as pathways such as the Ras signaling pathway, the *MAPK* signaling pathway, and bacterial invasion of epithelial cells.

One possible reason for the difference in miR-155 expression levels between our study and that of Srikok may be the use of skim milk instead of whole milk in the present study, which could explain the observed discrepancies in our data. This also highlights the importance of sample type when examining miRNA expression in milk or other body fluids for disease diagnosis. Moreover, previous studies have shown that miRNA expression profiles vary between lactating and non-lactating mammary glands [50], between mature milk and colostrum, and even across different organs (lung, brain, liver, and spleen) and whole blood [60]. Several other factors may account for the differences in miRNA expression observed in our study compared to previous research: the use of different sample types, such as milk, blood, monocytes, or mammary epithelial cells. The application of various laboratory techniques for profiling expression, such as NGS and real-time PCR, which can influence the specificity and sensitivity of the results as well [29,55]. In some studies, bacterial components were used to induce infection, and miRNA expression was examined at different time points [25,29]. In contrast, the present study and some similar investigations did not impose time constraints on the infection process [37]. Additionally, many studies did not specify the bacterial species responsible for mastitis, which may explain the differential expression of certain miRNAs depending on the specific pathogen involved.

Consistent with the studies conductedby Chen, Dilda, and Srikok, miR-223 expression was elevated in both clinical and subclinical mastitis in our study. Notably, the upregulation of miR-223 was significantly more pronounced in clinical mastitis caused by *E. coli* compared to subclinical mastitis associated with CoNS. Similarly, Pu et al. reported increased miR-223 expression in the mammary gland tissue of cows infected with *Streptococcus agalactiae* [61]. In contrast, Bagnicka reported a decrease in miR-223 expression in the mammary gland tissue of cattle infected with CoNS [62]. Moreover, Tzelos et al. found no statistically significant difference in miR-223 expression between mastitis and healthy cattle when comparing whole milk and skim milk samples [58]. Nevertheless, previous studies have suggested that miR-223 may serve as a novel biomarker for the diagnosis of bacterial mastitis [55, 63].

Consequently, our findings support the potential of miR-223 as a candidate biomarker for diagnosing clinical mastitis, given its expression in all cases of both clinical and subclinical mastitis, with significantly elevated levels in clinical mastitis. miR-223 is known to play a key role in the immune response during bovine mastitis [64], as well as in pulmonary and bacterial peritonitis infections [65]. According to Fang et al., bta-miR-223 acts as a key post-transcriptional regulator in the bovine mammary gland during S. aureus infection, where it controls the expression of pivotal innate immune genes like *CXCL14* [66]. Similarly, Tucker et al. emphasized the importance of miR-223 in regulating immune responses during mastitis infection [67]. Moreover, in humans, miR-223 has been identified as a specific and sensitive biomarker for the diagnosis of sepsis [68]. miR-223 also plays a critical role in the innate immune response during myeloid differentiation and in the function and activation of granulocytes, making it a key miRNA in infection and inflammation [62], and our bioinformatic screening suggests that it may also influence the *Ras* and *HIF-1* pathways through an unexpected association with *PLCG1* expression.

Bta-miR-223 is highly conserved across 15 different species without any changes in base pairing, and this evolutionary conservation underscores its importance and benefits to the host. Therefore, a therapeutic agent designed to target this miRNA could potentially be applicable across multiple species [67]. Similar to the findings of Lai et al. and Jadhav et al., we also observed an upregulation of miR-383 in the milk of cows with both clinical and subclinical mastitis; however, this increase was not statistically significant. Correspondingly, Jadhav et al. reported elevated expression of miR-383 in the milk of buffaloes with clinical mastitis [69]. Furthermore, it has been shown that the expression level of miR-383 increases in the RAW264.7 macrophage cell line stimulated with LPS [70]. The upregulation of miR-383 modulates the immune response by regulating genes such as *IL-1*, *TNF*, *COX-2*, *TLR4*, and *CXCL-1*, and the resulting tissue reaction to mammary gland bacterial infection in mastitis leads to inflammation [70]. Consequently, investigating the altered expression of miR-383 in milk and mammary tissue during mastitis provides insight into the mechanisms by which miRNAs regulate immune pathways [61]. Immune-related signaling pathways, such as *JAK-STAT*, *Ras*, and *AMPK*, are regulated by miR-383.

Other miRNAs investigated in our study were miR-200a and miR-205 in both clinical and subclinical mastitis. Compared to the healthy control group, miR-200a was significantly upregulated in clinical mastitis caused by *E. coli*. In subclinical mastitis caused by CoNS, miR-200a also showed upregulation relative to healthy controls.

Furthermore, miR-205 exhibited a non-significant upregulation in clinical mastitis compared to the healthy group, whereas it showed a significant downregulation in subclinical mastitis. In a study, Luoreng et al. demonstrated that miR-200a and miR-205 play important roles in the late immune stage of *E. coli* mastitis, and their expression levels were significantly elevated [71]. Our results are consistent with the findings of Luoreng et al.. In contrast, Li et al. reported a downregulation of miR-205 and miR-200b in mammary epithelial tissue infected with *staphylococcus aureus* [72]. This finding aligns with our observation of miR-205 expression in subclinical mastitis infected with CoNS, suggesting that the downregulation of miR-205 may be a common response to Gram-positive bacteria, independent of the specific species. The miR-200 family, which has been increasingly studied in recent years, is known to be upregulated in a wide range of diseases, a pattern that corroborates our results. Furthermore, Luoreng et al. demonstrated that bovine miR-200a regulates the development of *E. coli*-induced mastitis by targeting the bovine *ZEB1* gene [71]. One study demonstrated that miR-200a-3p expression is elevated in alcoholic hepatocytes and induces apoptosis by targeting the *ZEB2* gene [73]. Our bioinformatic analysis indicated that miR-200 is also involved in two key functional clusters: in cancer pathways by targeting genes such as *CDK6*, *ERBB2*, and *TGFBR2*, and in endocytosis by targeting genes including *PSD*, *GIT2, PDGFRA, WWP1*, and *TGFBR2*.

However, the association between miR-200a and mastitis in dairy cattle remains poorly understood, as current research in this area is limited and calls for more comprehensive and in-depth investigations. Likewise, the role of miR-205 in regulating the immune system has only been examined in a handful of studies [71]. Additional research indicates that miR-205 expression is elevated in individuals with allergic rhinitis [74], and that it modulates *erbB2*/*erbB3* expression in breast cancer cells, thereby facilitating apoptosis [75]. Notably, a miRNAomic analysis identified miR-205 as a candidate miRNA linked to mastitis resistance [64]. The miR-205 *COMMD1-NF-κB* signaling axis promotes the amplification of the inflammatory response [76]. Our bioinformatic findings indicated that by targeting key genes in major signaling pathways such as *Wnt, MAPK, FoxO*, and *p53*, miR-205 plays a significant role in modulating inflammatory responses during bovine mastitis.

Based on limited previous studies and our results, miR-200a could also serve as a biomarker for clinical mastitis caused by *E. coli* infection and be involved in the immune response in cattle, given that its expression changes were highly significant. However, elucidating its precise function in bovine mastitis will require further investigation. Another miRNA examined in our study was miR-92a. Its expression was significantly elevated in the milk of cows with clinical mastitis, whereas it was markedly reduced in the subclinical mastitis group. miR-92a plays a vital role in the host’s bacterial defense mechanisms in dairy cattle [77]. Additionally, miR-92a ranks among the most abundantly expressed miRNAs across different fractions of bovine milk, including milk fat, whey, and cellular components [78]. In a study, Casas et al. reported that miR-92a was downregulated in the serum of beef cattle infected with *Mycoplasma bovis* and suggested it as a potential biomarker [77]. Similarly, we observed downregulation of miR-92a in subclinical mastitis cases caused by coagulase-negative staphylococci (CoNS), despite differences in sample types and bacterial pathogens between the studies. Lia et al. further demonstrated that miR-92a is downregulated in Toll-like receptor (TLR)-expressing macrophages. Moreover, miR-92a modulates the production of pro-inflammatory cytokines such as *IL-6* and *TNF-α*; its overexpression suppresses these cytokines, while its inhibition leads to increased *IL-6* and *TNF-α* levels [79]. *TLRs* play a critical role in initiating immune responses and recognizing pathogens in the host [79]. Dysregulation of miR-92a is a recurrent feature in a spectrum of human malignancies, with documented aberrant expression in cancers of the lung, breast, stomach, prostate, colon, pancreas, liver, and kidney [80]. Lai et al. proposed miR-92a as a housekeeping gene for analysis of milk samples from mastitis cows, as its expression was stable in both healthy and mastitis animals [81]. However, research on miR-92a in cattle remains limited. Contrary to their findings, our analysis revealed that miR-92a expression differed significantly among the healthy, subclinical, and clinical mastitis groups. Our study encountered several limitations. A primary constraint was the single time-point sampling and the challenge of determining the precise onset of infection within the dairy herds. Most miRNAs are expressed differentially at various stages post-infection, reflecting their rapid temporal dynamics. Furthermore, many miRNAs induce relatively subtle changes in gene expression in response to infection, as they function as fine-tuners of gene regulation [82, 83]. Additionally, our sampling was conducted on cattle with natural infections, which may explain the discrepancies with studies employing *in vivo* experimental models. This inconsistency could be attributed to the potentially vastly different infectious agent doses in a natural setting. Consequently, future validation through a large-scale cohort study is essential to definitively assess the suitability of miRNAs as early diagnostic biomarkers for bovine mastitis.

## Conclusion

Analysis of miRNA expression patterns in mastitis not only holds great promise for early detection even before clinical symptoms appear, but also offers valuable insights into disease status and underlying causes. Moreover, the accessibility of non-invasive samples such as milk makes miRNAs especially attractive as valuable diagnostic biomarkers. The significant changes observed in miRNA expression levels in response to infections highlight their potential as early indicators of mastitis. This suggests that miRNAs could open a new frontier in mastitis diagnosis and/or prognosis. That said, validating these miRNAs as reliable diagnostic tools will require further investigation with larger sample sizes and diverse populations.

## Supporting information

S1 File (S1) contains all captions / descriptions of S1: Table 1 (Properties of primers for amplifying the uspA gene for E. coli), Table 2 (Properties of primers for amplifying the tuf gene for coagulase-negative staphylococci), Table 3 (Properties of primers for amplifying Mycoplasma genus), Table 4 (miRNA Expression Analysis; Average ΔCT, ΔΔCT, and Fold Change in Clinical Mastitis and Healthy Group), Table 5 (miRNA Expression Analysis; Average ΔCT, ΔΔCT, and Fold Change in Subclinical Mastitis and Healthy Group), Table 6 (A curated panel of predicted microRNA targets), Fig 1 (PCR amplification of uspA gene from milk samples in clinical mastitis), Fig 2 (PCR amplification of tuf genes from milk samples in subclinical mastitis), and Fig 3 (PCR amplification of Mycoplasma genus from milk samples in clinical, subclinical mastitis, and healthy control). (DOC)

## Abbreviations

TRAF 6: TNF receptor associated factor 6
IRAK 1: Interleukin 1 receptor associated kinase 1
TNF α: Tumor Necrosis Factor alpha
IL-6: Interleukin 6
NF-κB: Nuclear Factor Kappa-Light-Chain-Enhancer of Activated B Cells
mTOR: Mechanistic Target of Rapamycin
Ras: Rat Sarcoma Virus
MAPK: Mitogen-Activated Protein Kinase
JAK-STAT: Janus Kinase – Signal Transducer and Activator of Transcription
AMPK: AMP-Activated Protein Kinase
HIF-1: Hypoxia-Inducible Factor 1
p53: Tumor Protein p53
FOXO: Forkhead Box O
Wnt: Wingless-related integration site
TNFAIP3: TNF Alpha Induced Protein 3
Cox 2: Cyclooxygenase-2
CXCL-1: C-X-C motif chemokine ligand 1
TLR 4: Toll-like receptor 4
ZEB 1: Zinc finger E-box binding homeobox 1
COMMD 1: COMM domain-containing protein 1
CXCR 1: C-X-C motif chemokine receptor 1
CBL: Casitas B-lineage Lymphoma
RAC 1: Rac Family Small GTPase 1
PLCG 1: Phospholipase Cgamma1
CDK 6: Cell division protein kinase 6
ERBB 2: Erb-b2 receptor tyrosine kinase 2
TGFBR 2: Transforming growth factor beta receptor 2
PSD: Pleckstrin And Sec7 Domain Containing
GIT 2: G Protein-Coupled Receptor Kinase Interactor 2
PDGFRA: Platelet-derived growth factor receptor alpha
WWP 1: WW Domain Containing E3 Ubiquitin Protein Ligase 1

## References

[1] Hogeveen H, Huijps K, Lam TJGM. Economic aspects of mastitis: new developments. N Z Vet J. 2011;59(1):16–23. doi: 10.1080/00480169.2011.547165 21328153

[2] Seegers H, Fourichon C, Beaudeau F. Production effects related to mastitis and mastitis economics in dairy cattle herds. Vet Res. 2003;34(5):475–91. doi: 10.1051/vetres:2003027 14556691

[3] Gomes F, Saavedra MJ, Henriques M. Bovine mastitis disease/pathogenicity: evidence of the potential role of microbial biofilms. Pathog Dis. 2016;74(3):ftw006. doi: 10.1093/femspd/ftw006 26772653

[4] Ndahetuye JB, Persson Y, Nyman A-K, Tukei M, Ongol MP, Båge R. Aetiology and prevalence of subclinical mastitis in dairy herds in peri-urban areas of Kigali in Rwanda. Tropical Animal Health and Production. 2019;51(7):2037–44. 31030333 10.1007/s11250-019-01905-2 PMC6695354

[5] Kalińska A, Wójcik A, Slósarz J, Kruzińska B, Michalczuk M, Jaworski S. Occurrence and aetiology of staphylococcal mastitis-a review. Animal Science Papers & Reports. 2018;36(3).

[6] Detilleux J, Kastelic JP, Barkema HW. Mediation analysis to estimate direct and indirect milk losses due to clinical mastitis in dairy cattle. Prev Vet Med. 2015;118(4):449–56. doi: 10.1016/j.prevetmed.2015.01.009 25638330

[7] Zadoks RN, Middleton JR, McDougall S, Katholm J, Schukken YH. Molecular epidemiology of mastitis pathogens of dairy cattle and comparative relevance to humans. J Mammary Gland Biol Neoplasia. 2011;16(4):357–72. doi: 10.1007/s10911-011-9236-y 21968538 PMC3208832

[8] Vangroenweghe F, Lamote I, Burvenich C. Physiology of the periparturient period and its relation to severity of clinical mastitis. Domest Anim Endocrinol. 2005;29(2):283–93. doi: 10.1016/j.domaniend.2005.02.016 15950428

[9] Sutra L, Poutrel B. Virulence factors involved in the pathogenesis of bovine intramammary infections due to Staphylococcus aureus. J Med Microbiol. 1994;40(2):79–89. doi: 10.1099/00222615-40-2-79 8107066

[10] Oviedo-Boyso J, Valdez-Alarcón JJ, Cajero-Juárez M, Ochoa-Zarzosa A, López-Meza JE, Bravo-Patiño A, et al. Innate immune response of bovine mammary gland to pathogenic bacteria responsible for mastitis. J Infect. 2007;54(4):399–409. doi: 10.1016/j.jinf.2006.06.010 16882453

[11] M S, HA T. A Treatise on Bovine Mastitis: Disease and Disease Economics, Etiological Basis, Risk Factors, Impact on Human Health, Therapeutic Management, Prevention and Control Strategy. J Adv Dairy Res. 2015;04(01). doi: 10.4172/2329-888x.1000150

[12] Buitenhuis B, Røntved CM, Edwards SM, Ingvartsen KL, Sørensen P. In depth analysis of genes and pathways of the mammary gland involved in the pathogenesis of bovine Escherichia coli-mastitis. BMC Genomics. 2011;12:130. doi: 10.1186/1471-2164-12-130 21352611 PMC3053262

[13] Gilbert FB, Cunha P, Jensen K, Glass EJ, Foucras G, Robert-Granié C, et al. Differential response of bovine mammary epithelial cells to Staphylococcus aureus or Escherichia coli agonists of the innate immune system. Vet Res. 2013;44(1):40. doi: 10.1186/1297-9716-44-40 23758654 PMC3686618

[14] Bannerman DD, Paape MJ, Lee J-W, Zhao X, Hope JC, Rainard P. Escherichia coli and Staphylococcus aureus elicit differential innate immune responses following intramammary infection. Clin Diagn Lab Immunol. 2004;11(3):463–72. doi: 10.1128/CDLI.11.3.463-472.2004 15138171 PMC404560

[15] Lahouassa H, Moussay E, Rainard P, Riollet C. Differential cytokine and chemokine responses of bovine mammary epithelial cells to Staphylococcus aureus and Escherichia coli. Cytokine. 2007;38(1):12–21. doi: 10.1016/j.cyto.2007.04.006 17532224

[16] Viguier C, Arora S, Gilmartin N, Welbeck K, O’Kennedy R. Mastitis detection: current trends and future perspectives. Trends Biotechnol. 2009;27(8):486–93. doi: 10.1016/j.tibtech.2009.05.004 19616330

[17] Ruegg PL. A 100-Year Review: Mastitis Detection, Management, and Prevention. Journal of Dairy Science. 2017;100(12):10381–97. 29153171 10.3168/jds.2017-13023

[18] Liu W, Wang X. Prediction of functional microRNA targets by integrative modeling of microRNA binding and target expression data. Genome Biol. 2019;20(1):18. doi: 10.1186/s13059-019-1629-z 30670076 PMC6341724

[19] Steinkraus BR, Toegel M, Fulga TA. Tiny giants of gene regulation: experimental strategies for microRNA functional studies. Wiley Interdiscip Rev Dev Biol. 2016;5(3):311–62. doi: 10.1002/wdev.223 26950183 PMC4949569

[20] Ju Z, Jiang Q, Liu G, Wang X, Luo G, Zhang Y, et al. Solexa sequencing and custom microRNA chip reveal repertoire of microRNAs in mammary gland of bovine suffering from natural infectious mastitis. Anim Genet. 2018;49(1):3–18. doi: 10.1111/age.12628 29315680

[21] Eulalio A, Schulte L, Vogel J. The mammalian microRNA response to bacterial infections. RNA Biol. 2012;9(6):742–50. doi: 10.4161/rna.20018 22664920

[22] Lawless N, Foroushani ABK, McCabe MS, O’Farrelly C, Lynn DJ. Next generation sequencing reveals the expression of a unique miRNA profile in response to a gram-positive bacterial infection. PLoS One. 2013;8(3):e57543. doi: 10.1371/journal.pone.0057543 23472090 PMC3589390

[23] Deb R, Kumar A, Chakraborty S, Verma AK, Tiwari R, Dhama K, et al. Trends in diagnosis and control of bovine mastitis: a review. Pak J Biol Sci. 2013;16(23):1653–61. doi: 10.3923/pjbs.2013.1653.1661 24506032

[24] Weber JA, Baxter DH, Zhang S, Huang DY, Huang KH, Lee MJ, et al. The microRNA spectrum in 12 body fluids. Clin Chem. 2010;56(11):1733–41. doi: 10.1373/clinchem.2010.147405 20847327 PMC4846276

[25] Dilda F, Gioia G, Pisani L, Restelli L, Lecchi C, Albonico F, et al. Escherichia coli lipopolysaccharides and Staphylococcus aureus enterotoxin B differentially modulate inflammatory microRNAs in bovine monocytes. Vet J. 2012;192(3):514–6. doi: 10.1016/j.tvjl.2011.08.018 22033210

[26] Jin W, Ibeagha-Awemu EM, Liang G, Beaudoin F, Zhao X, Guan LL. Transcriptome microRNA profiling of bovine mammary epithelial cells challenged with Escherichia coli or Staphylococcus aureus bacteria reveals pathogen directed microRNA expression profiles. BMC Genomics. 2014;15:181. doi: 10.1186/1471-2164-15-181 24606609 PMC4029070

[27] Naeem A, Zhong K, Moisá SJ, Drackley JK, Moyes KM, Loor JJ. Bioinformatics analysis of microRNA and putative target genes in bovine mammary tissue infected with Streptococcus uberis. J Dairy Sci. 2012;95(11):6397–408. doi: 10.3168/jds.2011-5173 22959936

[28] Na RS, E GX, Sun W, Sun XW, Qiu XY, Chen LP, et al. Expressional analysis of immune-related miRNAs in breast milk. Genet Mol Res. 2015;14(3):11371–6. doi: 10.4238/2015.September.25.4 26436378

[29] Wang XP, Luoreng ZM, Zan LS, Raza SHA, Li F, Li N, et al. Expression patterns of miR-146a and miR-146b in mastitis infected dairy cattle. Mol Cell Probes. 2016;30(5):342–4. doi: 10.1016/j.mcp.2016.08.004 27531280

[30] Williams AE, Perry MM, Moschos SA, Larner-Svensson HM, Lindsay MA. Role of miRNA-146a in the regulation of the innate immune response and cancer. Biochem Soc Trans. 2008;36(Pt 6):1211–5. doi: 10.1042/BST0361211 19021527

[31] Wang XP, Luoreng ZM, Zan LS, Li F, Li N. Bovine miR-146a regulates inflammatory cytokines of bovine mammary epithelial cells via targeting the TRAF6 gene. Journal of Dairy Science. 2017;100(9):7648–58. 28690061 10.3168/jds.2017-12630

[32] Kim SW, Li Z, Moore PS, Monaghan AP, Chang Y, Nichols M, et al. A sensitive non-radioactive northern blot method to detect small RNAs. Nucleic Acids Res. 2010;38(7):e98. doi: 10.1093/nar/gkp1235 20081203 PMC2853138

[33] Li W, Ruan K. MicroRNA detection by microarray. Anal Bioanal Chem. 2009;394(4):1117–24. doi: 10.1007/s00216-008-2570-2 19132354

[34] Niu Y, Zhang L, Qiu H, Wu Y, Wang Z, Zai Y, et al. An improved method for detecting circulating microRNAs with S-Poly(T) Plus real-time PCR. Sci Rep. 2015;5:15100. doi: 10.1038/srep15100 26459910 PMC4602224

[35] Miao J, Wang J, Guo J, Gao H, Han K, Jiang C, et al. A plasmonic colorimetric strategy for visual miRNA detection based on hybridization chain reaction. Sci Rep. 2016;6:32219. doi: 10.1038/srep32219 27534372 PMC4989231

[36] Precazzini F, Detassis S, Imperatori AS, Denti MA, Campomenosi P. Measurements Methods for the Development of MicroRNA-Based Tests for Cancer Diagnosis. Int J Mol Sci. 2021;22(3):1176. doi: 10.3390/ijms22031176 33503982 PMC7865473

[37] Srikok S, Patchanee P, Boonyayatra S, Chuammitri P. Potential role of MicroRNA as a diagnostic tool in the detection of bovine mastitis. Prev Vet Med. 2020;182:105101. doi: 10.1016/j.prevetmed.2020.105101 32823253

[38] Lai Y-C, Fujikawa T, Maemura T, Ando T, Kitahara G, Endo Y, et al. Inflammation-related microRNA expression level in the bovine milk is affected by mastitis. PLoS One. 2017;12(5):e0177182. doi: 10.1371/journal.pone.0177182 28520748 PMC5435311

[39] Bergonier D, de Crémoux R, Rupp R, Lagriffoul G, Berthelot X. Mastitis of dairy small ruminants. Vet Res. 2003;34(5):689–716. doi: 10.1051/vetres:2003030 14556701

[40] Miller RH, Paape MJ, Acton JC. Comparison of milk somatic cell counts by Coulter and Fossomatic Counters. J Dairy Sci. 1986;69(7):1942–6. doi: 10.3168/jds.S0022-0302(86)80621-X 3745590

[41] Chen J, Griffiths MW. PCR differentiation of Escherichia coli from other gram-negative bacteria using primers derived from the nucleotide sequences flanking the gene encoding the universal stress protein. Lett Appl Microbiol. 1998;27(6):369–71. doi: 10.1046/j.1472-765x.1998.00445.x 9871356

[42] Hwang SM, Kim MS, Park KU, Song J, Kim E-C. Tuf gene sequence analysis has greater discriminatory power than 16S rRNA sequence analysis in identification of clinical isolates of coagulase-negative staphylococci. J Clin Microbiol. 2011;49(12):4142–9. doi: 10.1128/JCM.05213-11 21998419 PMC3232977

[43] Lierz M, Hagen N, Harcourt-Brown N, Hernandez-Divers SJ, Lüschow D, Hafez HM. Prevalence of mycoplasmas in eggs from birds of prey using culture and a genus-specific mycoplasma polymerase chain reaction. Avian Pathol. 2007;36(2):145–50. doi: 10.1080/03079450701213347 17479375

[44] Srikok S, Chuammitri P. MicroRNAs as a potential biomarker in bovine mastitis. Veterinary Integrative Sciences. 2016;14(1):1–12.

[45] Li R, Zhang C-L, Liao X-X, Chen D, Wang W-Q, Zhu Y-H, et al. Transcriptome microRNA profiling of bovine mammary glands infected with Staphylococcus aureus. Int J Mol Sci. 2015;16(3):4997–5013. doi: 10.3390/ijms16034997 25749476 PMC4394461

[46] Pu J, Li R, Zhang C, Chen D, Liao X, Zhu Y, et al. Expression profiles of miRNAs from bovine mammary glands in response to Streptococcus agalactiae-induced mastitis. J Dairy Res. 2017;84(3):300–8. doi: 10.1017/S0022029917000437 28831974

[47] Tili E, Michaille J-J, Cimino A, Costinean S, Dumitru CD, Adair B, et al. Modulation of miR-155 and miR-125b levels following lipopolysaccharide/TNF-alpha stimulation and their possible roles in regulating the response to endotoxin shock. J Immunol. 2007;179(8):5082–9. doi: 10.4049/jimmunol.179.8.5082 17911593

[48] Bian Y, Lei Y, Wang C, Wang J, Wang L, Liu L, et al. Epigenetic Regulation of miR-29s Affects the Lactation Activity of Dairy Cow Mammary Epithelial Cells. J Cell Physiol. 2015;230(9):2152–63. doi: 10.1002/jcp.24944 25656908

[49] Yuan Y, Tong L, Wu S. microRNA and NF-kappa B. microRNA: Basic Science: From Molecular Biology to Clinical Practice. Springer. 2015. p. 157–70.

[50] Li Z, Liu H, Jin X, Lo L, Liu J. Expression profiles of microRNAs from lactating and non-lactating bovine mammary glands and identification of miRNA related to lactation. BMC Genomics. 2012;13:731. doi: 10.1186/1471-2164-13-731 23270386 PMC3551688

[51] Livak KJ, Schmittgen TD. Analysis of relative gene expression data using real-time quantitative PCR and the 2(-Delta Delta C(T)) Method. Methods. 2001;25(4):402–8. doi: 10.1006/meth.2001.1262 11846609

[52] Dweep H, Gretz N, Sticht C. miRWalk database for miRNA–target interactions. RNA mapping: methods and protocols. Springer. 2014. p. 289–305. 10.1007/978-1-4939-1062-5_25 25055920

[53] Sherman BT, Hao M, Qiu J, Jiao X, Baseler MW, Lane HC, et al. DAVID: a web server for functional enrichment analysis and functional annotation of gene lists (2021 update). Nucleic acids research. 2022;50(W1):W216–W21. 10.1093/nar/gkac194 PMC9252805 35325185

[54] Lawless N, Vegh P, O’Farrelly C, Lynn DJ. The Role of microRNAs in Bovine Infection and Immunity. Front Immunol. 2014;5:611. doi: 10.3389/fimmu.2014.00611 25505900 PMC4245999

[55] Chen L, Liu X, Li Z, Wang H, Liu Y, He H, et al. Expression differences of miRNAs and genes on NF-κB pathway between the healthy and the mastitis Chinese Holstein cows. Gene. 2014;545(1):117–25. doi: 10.1016/j.gene.2014.04.071 24793582

[56] Ngo S, Moloney S, Li X, McNaughton L, Partridge A, Sheppard AM. Distinct microRNA signatures for mastitis measured in milk following natural exposure in dairy herds. Int J Anim Sci. 2017;1(1001):10.36876.

[57] Chuammitri P, Srikok S, Saipinta D, Boonyayatra S. The effects of quercetin on microRNA and inflammatory gene expression in lipopolysaccharide-stimulated bovine neutrophils. Vet World. 2017;10(4):403–10. doi: 10.14202/vetworld.2017.403-410 28507412 PMC5422244

[58] Tzelos T, Ho W, Charmana VI, Lee S, Donadeu FX. MiRNAs in milk can be used towards early prediction of mammary gland inflammation in cattle. Sci Rep. 2022;12(1):5131. doi: 10.1038/s41598-022-09214-9 35332227 PMC8948199

[59] Nahid MA, Pauley KM, Satoh M, Chan EKL. miR-146a Is Critical for Endotoxin-induced Tolerance. J Biological Chemistry. 2009;284(50):34590–9. doi: 10.1074/jbc.m109.056317 PMC2787321 19840932

[60] Hsieh C-H, Rau C-S, Jeng JC, Chen Y-C, Lu T-H, Wu C-J, et al. Whole blood-derived microRNA signatures in mice exposed to lipopolysaccharides. J Biomed Sci. 2012;19(1):69. doi: 10.1186/1423-0127-19-69 22849760 PMC3419134

[61] Pu J, Li R, Zhang C, Chen D, Liao X, Zhu Y, et al. Expression profiles of miRNAs from bovine mammary glands in response to Streptococcus agalactiae-induced mastitis. J Dairy Res. 2017;84(3):300–8. doi: 10.1017/S0022029917000437 28831974

[62] Bagnicka E, Kawecka-Grochocka E, Pawlina-Tyszko K, Zalewska M, Kapusta A, Kościuczuk E, et al. MicroRNA expression profile in bovine mammary gland parenchyma infected by coagulase-positive or coagulase-negative staphylococci. Vet Res. 2021;52(1):41. doi: 10.1186/s13567-021-00912-2 33676576 PMC7937231

[63] Sun J, Aswath K, Schroeder SG, Lippolis JD, Reinhardt TA, Sonstegard TS. MicroRNA expression profiles of bovine milk exosomes in response to Staphylococcus aureus infection. BMC Genomics. 2015;16(1). 10.1186/s12864-015-2044-9 PMC4609085 26475455

[64] Han S, Li X, Liu J, Zou Z, Luo L, Wu R, et al. Bta-miR-223 Targeting CBLB Contributes to Resistance to Staphylococcus aureus Mastitis Through the PI3K/AKT/NF-κB Pathway. Front Vet Sci. 2020;7:529. doi: 10.3389/fvets.2020.00529 33195489 PMC7475710

[65] Deny M, Romano M, Denis O, Casimir G, Chamekh M. Progressive Control of Streptococcus agalactiae-Induced Innate Inflammatory Response Is Associated with Time Course Expression of MicroRNA-223 by Neutrophils. Infect Immun. 2020;88(12):e00563-20. doi: 10.1128/IAI.00563-20 32958526 PMC7671897

[66] Fang L, Hou Y, An J, Li B, Song M, Wang X. Genome-wide transcriptional and post-transcriptional regulation of innate immune and defense responses of bovine mammary gland to Staphylococcus aureus. Front Cellular Infection Microbiol. 2016;6:193. 10.3389/fcimb.2016.00193 PMC5183581 28083515

[67] Tucker AR, Salazar NA, Ayoola AO, Memili E, Thomas BN, Morenikeji OB. Regulatory network of miRNA, lncRNA, transcription factor and target immune response genes in bovine mastitis. Sci Rep. 2021;11(1):21899. doi: 10.1038/s41598-021-01280-9 34753991 PMC8578396

[68] Wang JF, Yu ML, Yu G, Bian JJ, Deng XM, Wan XJ. Serum miR-146a and miR-223 as potential new biomarkers for sepsis. Biochemical and Biophysical Res Communications. 2010;394(1):184–8. 10.1016/j.bbrc.2010.02.145 20188071

[69] Jadhav AB, Ingole SD, Bharucha SV, Yoshitha KL, Gaikwad RV, Pharande RR, et al. Milk miRNA expression in buffaloes as a potential biomarker for mastitis. BMC Vet Res. 2024;20(1):150. doi: 10.1186/s12917-024-04002-1 38643124 PMC11031985

[70] Fan G, Jiang X, Wu X, Fordjour PA, Miao L, Zhang H, et al. Anti-Inflammatory Activity of Tanshinone IIA in LPS-Stimulated RAW264.7 Macrophages via miRNAs and TLR4-NF-κB Pathway. Inflammation. 2016;39(1):375–84. doi: 10.1007/s10753-015-0259-1 26639663

[71] Luoreng Z-M, Wang X-P, Mei C-G, Zan L-S. Expression profiling of peripheral blood miRNA using RNAseq technology in dairy cows with Escherichia coli-induced mastitis. Sci Rep. 2018;8(1):12693. doi: 10.1038/s41598-018-30518-2 30140010 PMC6107498

[72] Li L, Huang J, Zhang X, Ju Z, Qi C, Zhang Y, et al. One SNP in the 3’-UTR of HMGB1 gene affects the binding of target bta-miR-223 and is involved in mastitis in dairy cattle. Immunogenetics. 2012;64(11):817–24. doi: 10.1007/s00251-012-0641-1 22875364

[73] Zhao Y x, Sun Y y, Huang A l, Li X f, Huang C, Ma T t. MicroRNA-200a induces apoptosis by targeting ZEB2 in alcoholic liver disease. Cell Cycle. 2018;17(2):250–62. 29251244 10.1080/15384101.2017.1417708 PMC5884358

[74] Suojalehto H, Toskala E, Kilpeläinen M, Majuri ML, Mitts C, Lindström I. MicroRNA profiles in nasal mucosa of patients with allergic and nonallergic rhinitis and asthma. Int Forum Allergy Rhinol. 2013. 10.1002/alr.21179 23704072

[75] Wang S, Huang J, Lyu H, Lee C, Tan J, Wang J. Functional cooperation of miR-125a, miR-125b, and miR-205 in entinostat-induced downregulation of erbB2/erbB3 and apoptosis in breast cancer cells. Cell Death & Disease. 2013;4(3):e556-e. 10.1038/cddis.2013.79 PMC3615747 23519125

[76] Yeh D-W, Chen Y-S, Lai C-Y, Liu Y-L, Lu C-H, Lo J-F, et al. Downregulation of COMMD1 by miR-205 promotes a positive feedback loop for amplifying inflammatory- and stemness-associated properties of cancer cells. Cell Death Differ. 2016;23(5):841–52. doi: 10.1038/cdd.2015.147 26586569 PMC4832103

[77] Casas E, Cai G, Kuehn LA, Register KB, McDaneld TG, Neill JD. Association of MicroRNAs with Antibody Response to Mycoplasma bovis in Beef Cattle. PLoS One. 2016;11(8):e0161651. doi: 10.1371/journal.pone.0161651 27537842 PMC4990326

[78] Li R, Dudemaine P-L, Zhao X, Lei C, Ibeagha-Awemu EM. Comparative Analysis of the miRNome of Bovine Milk Fat, Whey and Cells. PLoS One. 2016;11(4):e0154129. doi: 10.1371/journal.pone.0154129 27100870 PMC4839614

[79] Lai L, Song Y, Liu Y, Chen Q, Han Q, Chen W, et al. MicroRNA-92a negatively regulates Toll-like receptor (TLR)-triggered inflammatory response in macrophages by targeting MKK4 kinase. J Biol Chem. 2013;288(11):7956–67. doi: 10.1074/jbc.M112.445429 23355465 PMC3597832

[80] Li M, Guan X, Sun Y, Mi J, Shu X, Liu F, et al. miR-92a family and their target genes in tumorigenesis and metastasis. Exp Cell Res. 2014;323(1):1–6. doi: 10.1016/j.yexcr.2013.12.025 24394541

[81] Lai YC, Fujikawa T, Ando T, Kitahara G, Koiwa M, Kubota C, et al. Rapid Communication: MiR-92a as a housekeeping gene for analysis of bovine mastitis-related microRNA in milk. J Anim Sci. 2017;95(6):2732–5. doi: 10.2527/jas.2017.1384 28727054

[82] Bartel DP. MicroRNAs: target recognition and regulatory functions. Cell. 2009;136(2):215–33. doi: 10.1016/j.cell.2009.01.002 19167326 PMC3794896

[83] O’Neill LA, Sheedy FJ, McCoy CE. MicroRNAs: the fine-tuners of Toll-like receptor signalling. Nat Rev Immunol. 2011;11(3):163–75. doi: 10.1038/nri2957 21331081

