# Supplementary material for: Investigation of certain miRNA expression levels in bovine mastitis cases caused by Escherichia coli and Coagulase-Negative Staphylococci
Source: PLoS One. 2026 Jul 29;21(7):e0352609. doi: 10.1371/journal.pone.0352609 (PMC13419229; doi:10.1371/journal.pone.0352609)
Supplement: S1 File — (DOC) [file pone.0352609.s001.doc]

**Investigation of certain miRNA expression levels in Bovine Mastitis cases caused by *Escherichia* *coli* and Coagulase-Negative Staphylococci**

**Author’s names:** Behnoush Khasheii, Pezhman Mahmoodi*, Taghi Zahraei Salehi, Abdolmajid Mohammadzadeh, Ali Sadeghi-nasab, Morteza Yavari

***Affiliation and e-mail address of the corresponding author:** Department of Pathobiology, Faculty of Veterinary Medicine, Bu-Ali Sina University, Hamedan, Iran.

**Email:** mahmoodi_pezhman@yahoo.com; mahmoodi_pezhman@basu.ac.ir

**Table 1:** Properties of primers for amplification of the *uspA* gene to identify *E. coli* cells*.*

| **Target gene** | **Amplicon (bp)** | **Sequence (5’→3’)** |
| --- | --- | --- |
| *uspA* | 884 bp | F: 5^׳^-CCGATACGCTGCCAATCAGT -3^׳^ |
|  |  | R: 5^׳^-ACGCAGACCGTAGGCCAGAT-3^׳^ |

| **Thermal stages of PCR** | | | |
| --- | --- | --- | --- |
| **Stage** | **Temperature** | **Time** | **Cycle** |
| Initial denaturation | 95^⸰c^ | 5 min | 1 |
| Denaturation  Annealing  extension | 94^⸰c^  58^⸰c^  72^⸰c^ | 30 sec  30 sec  1 min | 30 |
| Final extension | 72^⸰c^ | 5 min | 1 |

| Volume (µl) | Final concentration | Materials |
| --- | --- | --- |
| 10 µl | 1X | Taq DNA Polymerase Master Mix RED (Ampliqon/ Denmark) |
| 0.5 µl | 0.2 μM | Forward primer |
| 0.5 µl | 0.2 μM | Reverse primer |
| 1 µl | 50 ng | Template DNA |
| 8 µl | - | Distilled water |
| 20 µl | - | Total volume |


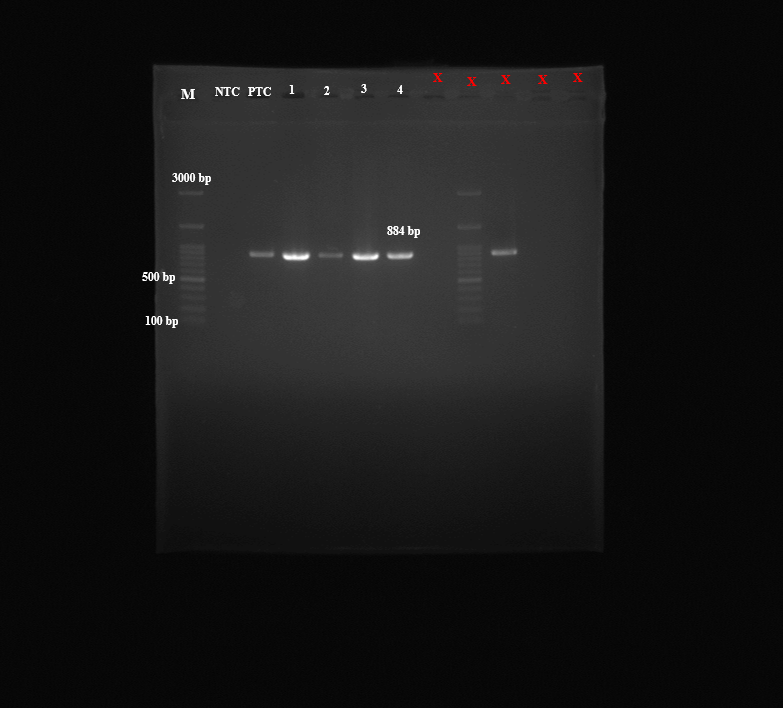


**Figure 1.** PCR amplification of *uspA* gene from milk samples in clinical mastitis; M: marker (100bp DNA ladder), NTC (negative control), PTC (positive control, *E. coli* ATCC 25922), lanes 1, 2, 3, 4, positive samples, X columns: not related to this study.

**100 bp**

**Table 2:** Properties of primers for amplification of the *tuf* gene for *coagulase-negative* staphylococci.

| **Target gene** | **Amplicon (bp)** | **Sequence (5’→3’)** |
| --- | --- | --- |
| *tuf* | 412 bp | F: 5^׳^-GCCAGTTGAGGACGTATTCT-3^׳^ |
|  |  | R: 5^׳^-CCATTTCAGTACCTTCTGGTAA-3^׳^ |

| **Thermal stages of PCR** | | | |
| --- | --- | --- | --- |
| **Stage** | **Temperature** | **Time** | **Cycle** |
| Initial denaturation | 95^⸰c^ | 15 min | 1 |
| Denaturation  Annealing  extension | 95^⸰c^  56^⸰c^  72^⸰c^ | 30 sec  30 sec  42 sec | 35 |
| Final extension | 72^⸰c^ | 10 min | 1 |

| Volume (µl) | Final concentration | Materials |
| --- | --- | --- |
| 10 µl | 1X | Taq DNA Polymerase Master Mix RED (Ampliqon/ Denmark) |
| 1 µl | 0.2 μM | Forward primer |
| 1 µl | 0.2 μM | Reverse primer |
| 2 µl | 50 ng | Template DNA |
| 6 µl | - | Distilled water |
| 20 µl | - | Total volume |


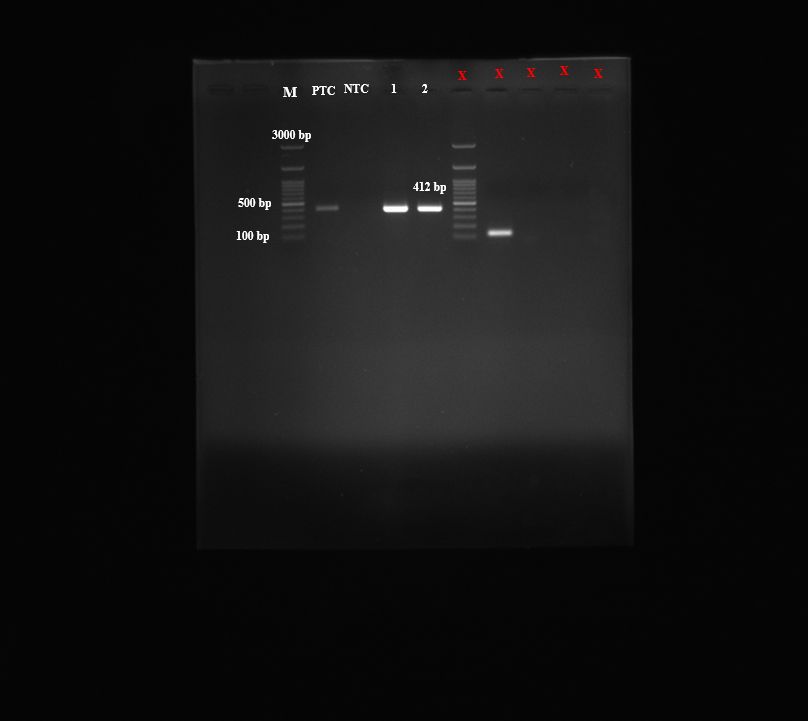


**Figure 2.** PCR amplification of *tuf* genes from milk samples in subclinical mastitis; M: marker (100bp DNA ladder), PTC (positive control, *Staphylococcus epidermidis* ATCC 12228), NTC (negative control), lanes 1, 2 positive samples, X columns: not related to this study.

**Table 3:** Properties of primers for the detection of *Mycoplasma* genus infection.

| Target gene | Amplicon (bp) | Sequence (5’→3’) |
| --- | --- | --- |
| ***GPF***  ***MGSO*** | 1013 bp | F: 5^׳^- GCTGGCTGTGTGCCTAATACA -3^׳^ |
|  |  | R: 5^׳^- TGCACCATCTGTCACTCTGTTAACCTC -3^׳^ |

| Thermal stages of PCR | | | |
| --- | --- | --- | --- |
| Stage | **Temperature** | **Time** | **Cycle** |
| Initial denaturation | 95^⸰c^ | 5 min | 1 |
| Denaturation  Annealing  extension | 94^⸰c^  58^⸰c^  72^⸰c^ | 1 min  1 min  1 min | 35 |
| Final extension | 72^⸰c^ | 10 min | 1 |

| Volume (µl) | Final concentration | Materials |
| --- | --- | --- |
| 10 µl | 1X | Taq DNA Polymerase Master Mix RED (Ampliqon/ Denmark) |
| 0.25 µl | 0.2 μM | Forward primer |
| 0.25 µl | 0.2 μM | Reverse primer |
| 5 µl | 50 ng | Template DNA |
| 4.5 µl | - | Distilled water |
| 20 µl | - | Total volume |


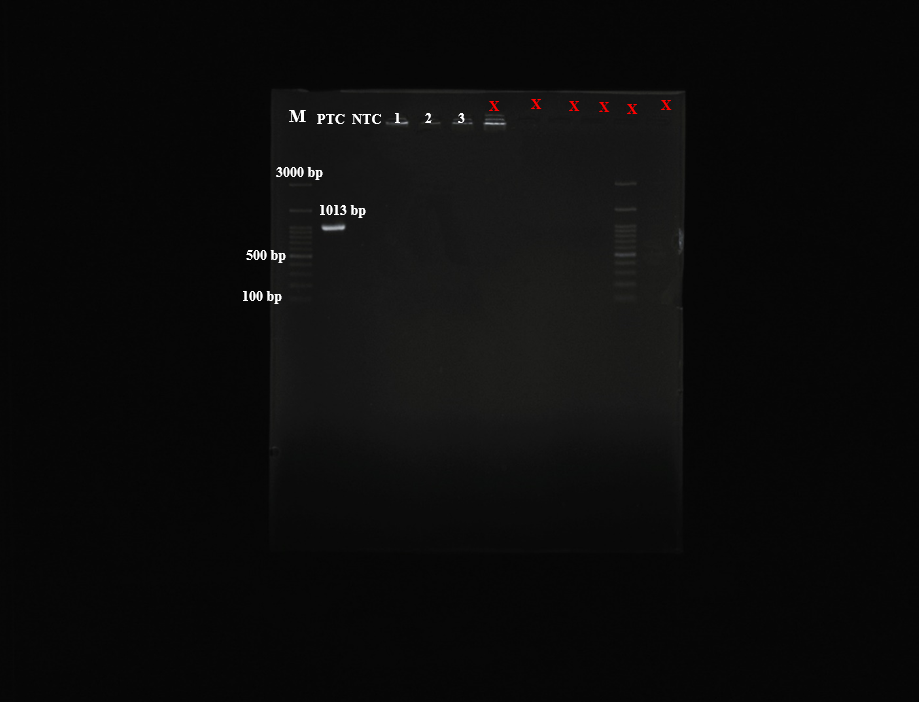


**Figure 3.** PCR detection of *Mycoplasma* genus from milk samples in clinical mastitis, subclinical mastitis, and healthy control groups; M: marker (100bp DNA ladder), PTC (positive control, *Mycoplasma* ATCC 10115), NTC (negative control), lanes 1, 2 , 3 negative samples, X columns: not related to this study.

**Table 4: miRNA Expression Analysis;** Average ΔCT, ΔΔCT, and Fold Change in Clinical Mastitis and Healthy Groups.

| Fold Change  2^- ΔΔCT^ | ΔΔCT | Mean of ΔCT (Mastitis) | Mean of ΔCT (Healthy) | Clinical Mastitis  (*E. coli*) |
| --- | --- | --- | --- | --- |
| 0.46 | 1.12 | -5.5 | -6.62 | miR-146a |
| 1.23 | -0.3 | -2.28 | -1.98 | miR-92a |
| 2.60 | -1.38 | -3.94 | -2.56 | miR-155 |
| 1.8 | -0.84 | -4.12 | -3.28 | miR-383 |
| 0.31 | 1.7 | 2.76 | 1.06 | miR-29B-2 |
| 8.93 | -3.16 | -3.16 | 0 | miR-223 |
| 0.37 | 1.42 | 1.34 | -0.08 | miR-148a |
| 47.84 | -5.58 | 7.08 | 12.66 | miR-200a |
| 2.50 | -1.32 | 5.12 | 6.44 | miR-205 |

**Table 5: miRNA Expression Analysis;** Average ΔCT, ΔΔCT, and Fold Change in Subclinical Mastitis and Healthy Groups.

| Fold Change  2^- ΔΔCT^ | ΔΔCT | Mean of ΔCT (Mastitis) | Mean of ΔCT (Healthy) | Subclinical Mastitis  (CoNS) |
| --- | --- | --- | --- | --- |
| 0.32 | 1.64 | -4.98 | -6.62 | miR-146a |
| 0.06 | 4.04 | 2.06 | -1.98 | miR-92a |
| 0.90 | 0.14 | -2.42 | -2.56 | miR-155 |
| 1.08 | -0.12 | -3.4 | -3.28 | miR-383 |
| 0.19 | 2.42 | 3.48 | 1.06 | miR-29B-2 |
| 3.89 | -1.96 | -1.96 | 0 | miR-223 |
| 0.23 | 2.12 | 2.04 | -0.08 | miR-148a |
| 5.81 | -2.54 | 10.12 | 12.66 | miR-200a |
| 0.43 | 1.2 | 7.64 | 6.44 | miR-205 |

**Table 6: A curated panel of predicted microRNA targets**.

| **miRNA** | **KEGG pathway name** | **Target Genes** |
| --- | --- | --- |
| **bta-miR-146a** | mTOR signaling pathway | *SLC7A5, FZD3, RRAGD, FNIP2, LPIN2* |
|  | Butanoate metabolism | *SLC7A5, FZD3, RRAGD, FNIP2, LPIN2* |
|  | Metabolic pathways | *CA12, ELOVL1, GLO1, ENTPD6, ABAT, ACSM4, SYNJ2, MTMR4, PTDSS2, DPM2, ATP6V0A4, L2HGDH, RDH13, ENOPH1, LPIN2, HMGCLL1, EARS2, PNLIPRP3* |
| **bta-miR-92a** | Terpenoid backbone biosynthesis | *MVK, HMGCS1, FNTB, HMGCR, DHDDS* |
|  | Sphingolipid metabolism | *NEU2, NEU3, ACER1, UGT8, ASAH1, CERS6, SPTLC3, SGMS2* |
|  | C-type lectin receptor signaling pathway | *NFKBIA, MAPK9, CYLD, PAK1, ARHGEF12, CCL22, SYK, NLRP3, NFATC2, PTPN11, IKBKE* |
|  | Regulation of actin cytoskeleton | *ARHGEF12, ITGB5, PDGFA, SSH2, FGF1, C8A, EGFR, MYL12A, ARHGAP35, MYLK4, PAK1, CXCL12, SPATA13, PIP5K1A, ITGB7, EZR, PPP1R12B, PFN2* |
|  | Focal adhesion | *VASP, ITGB5, PDGFA, PARVA, EGFR, MYL12A, ARHGAP35, MYLK4, MAPK9, PAK1, RAPGEF1, KDR, PIP5K1A, ITGB7, FLNB, PPP1R12B* |
|  | Nucleotide metabolism | *CDA, RRM1, ENTPD2, NME6, NME7, CMPK1, UPP2, TK2, ADA* |
|  | Metabolic pathways | *CDA, PLA2G2D1, GALNT14, PGAP2, GALNT16, UXS1, POMT1, DBH, PLA2G2D4, SAT1, SGSH, CA2, SPTLC3, UPP2, TK2, ME3, AASS, BCKDHA, ACAD8, GUCY1A1, ALG9, ENTPD2, CERS6, HMGCS1, DNMT3A, SIRT4, ACOD1, SDHC, LOC530929, SIRT1, INPP4A, HADHB, NME6, NME7, ITPKA, CMPK1, UQCRC1, B3GNT3, IDO2, BCAT1, BLVRA, DGKI, PAFAH1B1, IDO1, PIGH, PRPS2, GCDH, ASAH1, PFKFB3, MVK, ODC1, AKR1D1, DOT1L, HSD17B3, HMGCR, SGMS2, FUT1, NEU2, NEU3, UGT8, MAN2A1, CBS, CHST10, SMYD1, INPP5K, PIP5K1A, ST3GAL1, CBR4, AOC3, RRM1, GSTM2, TMEM86B, LOC100139075, PLA2G2C, GATB, B3GALT6, NUDT9, ACER1, PPT1, ECHDC1, PEMT, NAGS, PPT2, LPIN1, PFKM, ADA, EZH1* |
|  | Endocytosis | *SMAD2, RAB4A, IQSEC2, PML, EGFR, EPN2, RNF41, CYTH3, SNX4, ZFYVE27, CAPZA1, CHMP4B, PIP5K1A, AMPH, CHMP7, VPS36, VPS25, RAB8A* |
|  | AMPK signaling pathway | *PFKFB3, CAB39L, CREB3L2, PPP2R5D, HMGCR, PPP2R5C, ADRA1A, SIRT1, PFKM, RAB8A, CREB5* |
| **bta-miR-155** | ErbB signaling pathway | *ABL2, BRAF, CBL* |
|  | Bacterial invasion of epithelial cells | *SHC4, RAC1, CBL, SEPTIN8* |
|  | Ras signaling pathway | *PDGFRB, SHC4, NTF3, ABL2, RAC1* |
|  | MAPK signaling pathway | *PDGFRB, MAP3K2, NTF3, RAC1, MAP3K13* |
|  | Adherens junction | *FER, CSNK2A2, RAC1* |
|  | Choline metabolism in cancer | *PDGFRB, SP1, RAC1* |
|  | pathways in cancer | *PDGFRB, PTGER1, SP1, PTGER2, RAC1, CBL* |
|  | Neuroactive ligand-receptor interaction | *GABBR2, CYSLTR2, GLRB, PTGER1, PTGER2* |
|  | Human cytomegalovirus infection | *PTGER1, SP1, PTGER2, RAC1* |
|  | Neurotrophin signaling pathway | *SHC4, NTF3, RAC1* |
| **bta-miR-383** | Oxytocin signaling pathway | *KCNJ5, GUCY1A1, PRKAB2, PLA2G4E, RAF1, PRKAB1, KCNJ2, CAMKK2* |
|  | JAK-STAT signaling pathway | *LOC523509, IFNGR1, IL10RA, LEPR, STAT4, IL31RA, LOC526769, RAF1, PRLR, IFNAR1* |
|  | Ras signaling pathway | *PLA2G2F, BRAP, PLA2G4E, INSR, LOC615045, FOXO4, LOC526769, RAF1, PLD1, FGFR3, PLD2* |
|  | AMPK signaling pathway | *PFKFB4, PRKAB2, PFKFB3, INSR, LEPR, PRKAB1, CREB5, CAMKK2* |
|  | Metabolic pathways | *LOC530653, INPPL1, EHMT1, PIGW, PFAS, MECOM, ME1, ENPP6, COLGALT1, EARS2, XYLB, MCCC2, VKORC1, GUCY1A1, CERS5, DGAT2, ATP6V1G2, PLA2G4E, ACSL1, GPT2, ALG11, KMO, GANC, B3GNT6, LOC615045, DPYD, DGKI, PAFAH1B1, PFKFB4, PFKFB3, ASAH2, MTMR3, MVK, GMPS, SGMS2, PLD1, PLD3, PLD2, GANAB, INPP5A, MAN2A2, MAN2A1, ST3GAL6, XDH, NADK, FKTN, PLA2G2F, AGK, GATB, FMO5, NUDT9, DHRS9, GLB1* |
|  | Fc gamma R-mediated phagocytosis | *ACTR3, SYK, PLA2G4E, INPPL1, RAF1, PLD1, WASF2, PLD2* |
|  | Endocytosis | *ACTR3, ARFGEF2, GIT2, AGAP2, VPS26A, CBL, PLD1, PLD2, RAB31, PSD2, CXCR1, VPS45, FGFR3, RAB11FIP4* |
| **miR-29B-2** | Chemokine signaling pathway | *SHC3, CXCR1, GNB4* |
|  | Endocytosis | *CXCR1, CHMP1A, VPS36, FGFR2* |
| **bta-miR-223** | Phosphatidylinositol signaling system | *INPP4A, MTMR2, ITPR1, PLCG2* |
|  | Pathways in cancer | *HEYL, EGLN2, NOS2, COL4A2, TXNRD1, PLCG2, WNT9A, IL12RB1, SOS2, RELA* |
|  | C-type lectin receptor signaling pathway | *KSR1, ITPR1, PLCG2, RELA* |
|  | Ras signaling pathway | *EFNA3, KSR1, RASGRF2, PLCG2, SOS2, RELA* |
|  | HIF-1 signaling pathway | *EGLN2, NOS2, PLCG2, RELA* |
|  | Metabolic pathways | *KMT2D, MTMR2, NOS2, GALNT15, PGAP4, POMT1, NAGA, GATB, GNS, ENO4, PLD3, NUDT9, INPP4A, ADI1, SARDH, PLCG2, ACSS1, LPIN3, EARS2, AASS, PAFAH1B1* |
|  | Calcium signaling pathway | *NOS2, ITPR1, PLCG2, TACR2, ATP2A2, ATP2B1* |
| **bta-miR-148a** | Regulation of actin cytoskeleton | *ACTR3, APC, ARPC5L, ITGAE, FGF1* |
|  | Breast cancer | *APC, E2F1, BAK1, FGF1* |
| **bta-miR-200a** | Phosphonate and phosphinate metabolism | *PCYT1B, SELENOI* |
|  | Endocytosis | *PSD, GIT2, PDGFRA, WWP1, TGFBR2* |
|  | Central carbon metabolism in cancer | *PDGFRA, PDHA1, ERBB2* |
|  | Pancreatic cancer | *CDK6, ERBB2, TGFBR2* |
| **bta-miR-205** | Phospholipase D signaling pathway | *AGPAT5, PTGFR, ARF2, SYK, SPHK1, F2R, TSC1, DGKZ, GRM1, DNM1, PIK3R5, CYTH3, MRAS, GRM6, AKT2, GNAS, PLCB1, DGKI, ARF6* |
|  | Glycerophospholipid metabolism | *AGPAT5, PLA2G2D1, PLA2G12A, PCYT1A, SAMD8, CHAT, MBOAT1, LPCAT1, PLA2G3, PLA2G2D4, DGKZ, LCLAT1, PHOSPHO1, DGKI* |
|  | p53 signaling pathway | *CASP9, GADD45B, SESN3, APAF1, CCNG2, PERP, SESN1, SESN2, PMAIP1, THBS1, SERPINB5, TP73* |
|  | FoxO signaling pathway | *SMAD4, PRKAB2, CDKN1B, SMAD3, GADD45B, TGFB3, PLK2, NLK, KLF2, GRM1, PRKAG3, AKT2, CCNG2, CAT, S1PR1, SKP2* |
|  | Wnt signaling pathway | *TLE4, SMAD4, WNT10A, FZD2, SMAD3, WNT3A, CAMK2A, PRICKLE2, PSEN1, NLK, WNT16, VANGL2, APC, DAAM2, RSPO2, ROR1, PLCB1* |
|  | Endocytosis | *ARF2, SMAD3, IQSEC2, AP2A1, ARFGAP3, DNM1, CDC42, CYTH3, EHD2, RABEP1, RAB31, PSD2, KIF5C, PIP5KL1, RAB35, CHMP4B, LDLR, SH3GL2, ARF5, RAB11FIP4, ARF6* |
|  | cAMP signaling pathway | *RYR2, ADCYAP1R1, PTCH1, F2R, CAMK2A, ATP2B4, ATP2B3, ATP1B4, ATP2A2, ATP2B2, CRHR2, VAV2, NFKBIA, LIPE, AKT2, GNAS, ADORA1, MYL9, BVES, CREB5* |
|  | AMPK signaling pathway | *PFKFB2, LIPE, PRKAB2, EEF2K, SCD, LEP, AKT2, TSC1, EEF2, ADRA1A, PRKAG3, CREB5* |
|  | MAPK signaling pathway | *DUSP2, GADD45B, TGFB3, CACNA2D2, NLK, EFNA5, DUSP9, VEGFA, DUSP7, CDC42, MRAS, MAPKAPK3, TAOK3, AKT2, RAPGEF2, FLNA, MAP3K9, MAP3K6, MAP3K13, PTPN5, MAP4K4, MAP3K12* |
|  | Cell adhesion molecules | *NLGN3, NTNG2, NLGN2, SDC3, F11R, SELE, VCAN, MPZ, CLDN12, CD58, TIGIT, NECTIN3, CD34, ICOSLG* |
|  | Platelet activation | *PTGIR, SYK, AKT2, ITGB3, SNAP23, F2R, GNAS, BTK, TLN2, PLCB1, PIK3R5* |
|  | Pathways in cancer | *CDKN1B, PTGER1, CAMK2A, ETS1, CDC42, CASP9, AKT2, E2F1, PMAIP1, E2F3, SKP2, JAK3, IL13RA1, NKX3-1, ARNT2, SMAD4, WNT10A, FZD2, SMAD3, IL4R, GADD45B, NOS2, APAF1, TGFB3, WNT3A, TXNRD1, PTCH1, STAT2, F2R, WNT16, VEGFA, NFKBIA, APC, GNAS, PLCB1* |

**Abbreviations**:

uspA: Universal stress protein A

tuf: Elongation factor Tu

**SLC7A5**: Solute Carrier Family 7 Member

**FZD3**: Frizzled Class Receptor 3

**RRAGD**: Ras-Related GTP Binding D

**FNIP2**: Folliculin Interacting Protein 2

**LPIN2**: Lipin 2

CA12: Carbonic Anhydrase 12

ELOVL1: ELOVL Fatty Acid Elongase 1

GLO1: Glyoxalase 1

ENTPD6: Ectonucleoside Triphosphate Diphosphohydrolase 6

ABAT: 4-Aminobutyrate Aminotransferase

ACSM4: Acyl-CoA Synthetase Medium-Chain Family Member 4

SYNJ2: Synaptojanin 2

MTMR4: Myotubularin Related Protein 4

PTDSS2: Phosphatidylserine Synthase 2

DPM2: Dolichyl-Phosphate Mannosyltransferase Subunit 2

ATP6V0A4: ATPase H+ Transporting V0 Subunit A4

L2HGDH: L-2-Hydroxyglutarate Dehydrogenase

RDH13: Retinol Dehydrogenase 13

ENOPH1: Enolase-Phosphatase 1

HMGCLL1: 3-Hydroxymethyl-3-Methylglutaryl-CoA Lyase Like 1

EARS2: Glutamyl-tRNA Synthetase 2, Mitochondrial

PNLIPRP3: Pancreatic Lipase Related Protein 3

MVK: Mevalonate Kinase

HMGCS1: 3-Hydroxy-3-Methylglutaryl-CoA Synthase 1

FNTB: Farnesyltransferase Subunit Beta

HMGCR: 3-Hydroxy-3-Methylglutaryl-CoA Reductase

DHDDS: Dehydrodolichyl Diphosphate Synthase

**ARHGEF12**: Rho Guanine Nucleotide Exchange Factor 12

**ITGB5**: Integrin Subunit Beta 5

**PDGFA**: Platelet Derived Growth Factor Subunit A

**SSH2**: Slingshot Protein Phosphatase 2

**FGF1**: Fibroblast Growth Factor 1

**C8A**: Complement C8 Alpha Chain

**EGFR**: Epidermal Growth Factor Receptor

**MYL12A**: Myosin Light Chain 12A

**ARHGAP35**: Rho GTPase Activating Protein 35

**MYLK4**: Myosin Light Chain Kinase 4

**PAK1**: P21 (RAC1) Activated Kinase 1

**CXCL12**: C-X-C Motif Chemokine Ligand 12

**SPATA13**: Spermatogenesis Associated 13

**PIP5K1A**: Phosphatidylinositol-4-Phosphate 5-Kinase Type 1 Alpha

**ITGB7**: Integrin Subunit Beta 7

**EZR**: Ezrin

**PPP1R12B**: Protein Phosphatase 1 Regulatory Subunit 12B

**PFN2**: Profilin 2

VASP: Vasodilator-Stimulated Phosphoprotein

ITGB5: Integrin Subunit Beta 5

PDGFA: Platelet Derived Growth Factor Subunit A

PARVA: Parvin Alpha

EGFR: Epidermal Growth Factor Receptor

MYL12A: Myosin Light Chain 12A

ARHGAP35: Rho GTPase Activating Protein 35

MYLK4: Myosin Light Chain Kinase 4

MAPK9: Mitogen-Activated Protein Kinase 9

PAK1: P21 (RAC1) Activated Kinase 1

RAPGEF1: Rap Guanine Nucleotide Exchange Factor 1

KDR: Kinase Insert Domain Receptor

PIP5K1A: Phosphatidylinositol-4-Phosphate 5-Kinase Type 1 Alpha

ITGB7: Integrin Subunit Beta 7

FLNB: Filamin B

PPP1R12B: Protein Phosphatase 1 Regulatory Subunit 12B

**CDA**: Cytidine Deaminase

**RRM1**: Ribonucleotide Reductase Catalytic Subunit M1

**ENTPD2**: Ectonucleoside Triphosphate Diphosphohydrolase 2

**NME6**: NME/NM23 Family Member 6

**NME7**: NME/NM23 Family Member 7

**CMPK1**: UMP-CMP Kinase 1 (or Cytidine Monophosphate Kinase 1)

**UPP2**: Uridine Phosphorylase 2

**TK2**: Thymidine Kinase 2

**ADA**: Adenosine Deaminase

**CDA**: Cytidine Deaminase

**PLA2G2D1**: Phospholipase A2 Group IID Member 1

**GALNT14**: Polypeptide N-Acetylgalactosaminyltransferase 14

**PGAP2**: Post-GPI Attachment to Proteins 2

**GALNT16**: Polypeptide N-Acetylgalactosaminyltransferase 16

**UXS1**: UDP-Glucuronate Decarboxylase 1

**POMT1**: Protein O-Mannosyltransferase 1

**DBH**: Dopamine Beta-Hydroxylase

**PLA2G2D4**: Phospholipase A2 Group IID Member 4

**SAT1**: Spermidine/Spermine N1-Acetyltransferase 1

**SGSH**: N-Sulfoglucosamine Sulfohydrolase

**CA2**: Carbonic Anhydrase 2

**SPTLC3**: Serine Palmitoyltransferase Long Chain Base Subunit 3

**UPP2**: Uridine Phosphorylase 2

**TK2**: Thymidine Kinase 2

**ME3**: Malic Enzyme 3

**AASS**: Aminoadipate-Semialdehyde Synthase

**BCKDHA**: Branched Chain Keto Acid Dehydrogenase E1 Subunit Alpha

**ACAD8**: Acyl-CoA Dehydrogenase Family Member 8

**GUCY1A1**: Guanylate Cyclase 1 Soluble Subunit Alpha 1

**ALG9**: ALG9 Alpha-1,2-Mannosyltransferase

**ENTPD2**: Ectonucleoside Triphosphate Diphosphohydrolase 2

**CERS6**: Ceramide Synthase 6

**HMGCS1**: 3-Hydroxy-3-Methylglutaryl-CoA Synthase 1

**DNMT3A**: DNA Methyltransferase 3 Alpha

**SIRT4**: Sirtuin 4

**ACOD1**: Aconitate Decarboxylase 1

**SDHC**: Succinate Dehydrogenase Complex Subunit C

**LOC530929**: Uncharacterized LOC530929 (note: this is a locus identifier, not a named gene)

**SIRT1**: Sirtuin 1

**INPP4A**: Inositol Polyphosphate-4-Phosphatase Type I A

**HADHB**: Hydroxyacyl-CoA Dehydrogenase Trifunctional Multienzyme Complex Subunit Beta

**NME6**: NME/NM23 Family Member 6

**NME7**: NME/NM23 Family Member 7

**ITPKA**: Inositol-Trisphosphate 3-Kinase A

**CMPK1**: UMP-CMP Kinase 1 (Cytidine Monophosphate Kinase 1)

**UQCRC1**: Ubiquinol-Cytochrome C Reductase Core Protein 1

**B3GNT3**: UDP-GlcNAc:BetaGal Beta-1,3-N-Acetylglucosaminyltransferase 3

**IDO2**: Indoleamine 2,3-Dioxygenase 2

**BCAT1**: Branched Chain Amino Acid Transaminase 1

**BLVRA**: Biliverdin Reductase A

**DGKI**: Diacylglycerol Kinase Iota

**PAFAH1B1**: Platelet Activating Factor Acetylhydrolase 1b Regulatory Subunit 1

**IDO1**: Indoleamine 2,3-Dioxygenase 1

**PIGH**: Phosphatidylinositol Glycan Anchor Biosynthesis Class H

**PRPS2**: Phosphoribosyl Pyrophosphate Synthetase 2

**GCDH**: Glutaryl-CoA Dehydrogenase

**ASAH1**: N-Acylsphingosine Amidohydrolase 1 (Acid Ceramidase)

**PFKFB3**: 6-Phosphofructo-2-Kinase/Fructose-2,6-Biphosphatase 3

**MVK**: Mevalonate Kinase

**ODC1**: Ornithine Decarboxylase 1

**AKR1D1**: Aldo-Keto Reductase Family 1 Member D1

**DOT1L**: DOT1 Like Histone Lysine Methyltransferase

**HSD17B3**: Hydroxysteroid 17-Beta Dehydrogenase 3

**HMGCR**: 3-Hydroxy-3-Methylglutaryl-CoA Reductase

**SGMS2**: Sphingomyelin Synthase 2

**FUT1**: Fucosyltransferase 1

**NEU2**: Neuraminidase 2

**NEU3**: Neuraminidase 3

**UGT8**: UDP-Glycosyltransferase 8

**MAN2A1**: Mannosidase Alpha Class 2A Member 1

**CBS**: Cystathionine Beta-Synthase

**CHST10**: Carbohydrate Sulfotransferase 10

**SMYD1**: SET and MYND Domain Containing 1

**INPP5K**: Inositol Polyphosphate-5-Phosphatase K

**PIP5K1A**: Phosphatidylinositol-4-Phosphate 5-Kinase Type 1 Alpha

**ST3GAL1**: ST3 Beta-Galactoside Alpha-2,3-Sialyltransferase 1

**CBR4**: Carbonyl Reductase 4

**AOC3**: Amine Oxidase Copper Containing 3

**RRM1**: Ribonucleotide Reductase Catalytic Subunit M1

**GSTM2**: Glutathione S-Transferase Mu 2

**TMEM86B**: Transmembrane Protein 86B

**LOC100139075**: Uncharacterized LOC100139075 (locus identifier)

**PLA2G2C**: Phospholipase A2 Group IIC

**GATB**: Glutamyl-tRNA Amidotransferase Subunit B

**B3GALT6**: Beta-1,3-Galactosyltransferase 6

**NUDT9**: Nudix Hydrolase 9

**ACER1**: Alkaline Ceramidase 1

**PPT1**: Palmitoyl-Protein Thioesterase 1

**ECHDC1**: Enoyl-CoA Hydratase Domain Containing 1

**PEMT**: Phosphatidylethanolamine N-Methyltransferase

**NAGS**: N-Acetylglutamate Synthase

**PPT2**: Palmitoyl-Protein Thioesterase 2

**LPIN1**: Lipin 1

**PFKM**: Phosphofructokinase, Muscle

**ADA**: Adenosine Deaminase

**EZH1**: Enhancer of Zeste 1 Polycomb Repressive Complex 2 Subunit

**ABL2**: ABL Proto-Oncogene 2, Non-Receptor Tyrosine Kinase

**BRAF**: B-Raf Proto-Oncogene, Serine/Threonine Kinase

**CBL**: Cbl Proto-Oncogene

**SHC4**: SHC Adaptor Protein 4

**RAC1**: Rac Family Small GTPase 1

**SEPTIN8**: Septin 8

**PDGFRB**: Platelet Derived Growth Factor Receptor Beta

**NTF3**: Neurotrophin 3

**MAP3K2**: Mitogen-Activated Protein Kinase Kinase Kinase 2

**MAP3K13**: Mitogen-Activated Protein Kinase Kinase Kinase 13

**FER**: FER Tyrosine Kinase

**CSNK2A2**: Casein Kinase 2 Alpha 2

**SP1**: Sp1 Transcription Factor

**PTGER1**: Prostaglandin E Receptor 1

**PTGER2**: Prostaglandin E Receptor 2

**GABBR2**: Gamma-Aminobutyric Acid Type B Receptor Subunit 2

**CYSLTR2**: Cysteinyl Leukotriene Receptor 2

**GLRB**: Glycine Receptor Beta

**KCNJ5**: Potassium Inwardly Rectifying Channel Subfamily J Member 5

**GUCY1A1**: Guanylate Cyclase 1 Soluble Subunit Alpha 1

**PRKAB2**: Protein Kinase AMP-Activated Non-Catalytic Subunit Beta 2

**PLA2G4E**: Phospholipase A2 Group IVE

**RAF1**: Raf-1 Proto-Oncogene, Serine/Threonine Kinase

**PRKAB1**: Protein Kinase AMP-Activated Non-Catalytic Subunit Beta 1

**KCNJ2**: Potassium Inwardly Rectifying Channel Subfamily J Member 2

**CAMKK2**: Calcium/Calmodulin Dependent Protein Kinase Kinase 2

**LOC523509**: Uncharacterized LOC523509 (locus identifier)

**IFNGR1**: Interferon Gamma Receptor 1

**IL10RA**: Interleukin 10 Receptor Subunit Alpha

**LEPR**: Leptin Receptor

**STAT4**: Signal Transducer and Activator of Transcription 4

**IL31RA**: Interleukin 31 Receptor Subunit Alpha

**LOC526769**: Uncharacterized LOC526769 (locus identifier)

**PRLR**: Prolactin Receptor

**IFNAR1**: Interferon Alpha and Beta Receptor Subunit 1

**PLA2G2F**: Phospholipase A2 Group IIF

**BRAP**: BRCA1 Associated Protein

**INSR**: Insulin Receptor

**LOC615045**: Uncharacterized LOC615045 (locus identifier)

**FOXO4**: Forkhead Box O4

**PLD1**: Phospholipase D1

**FGFR3**: Fibroblast Growth Factor Receptor 3

**PLD2**: Phospholipase D2

**PFKFB4**: 6-Phosphofructo-2-Kinase/Fructose-2,6-Biphosphatase 4

**PFKFB3**: 6-Phosphofructo-2-Kinase/Fructose-2,6-Biphosphatase 3

**CREB5**: cAMP Responsive Element Binding Protein 5

**LOC530653**: Uncharacterized LOC530653 (locus identifier)

**INPPL1**: Inositol Polyphosphate Phosphatase Like 1

**EHMT1**: Euchromatic Histone Lysine Methyltransferase 1

**PIGW**: Phosphatidylinositol Glycan Anchor Biosynthesis Class W

**PFAS**: Phosphoribosylformylglycinamidine Synthase

**MECOM**: MDS1 and EVI1 Complex Locus

**ME1**: Malic Enzyme 1

**ENPP6**: Ectonucleotide Pyrophosphatase/Phosphodiesterase 6

**COLGALT1**: Collagen Beta(1-O)Galactosyltransferase 1

**EARS2**: Glutamyl-tRNA Synthetase 2, Mitochondrial

**XYLB**: Xylulokinase

**MCCC2**: Methylcrotonoyl-CoA Carboxylase 2

**VKORC1**: Vitamin K Epoxide Reductase Complex Subunit 1

**GUCY1A1**: Guanylate Cyclase 1 Soluble Subunit Alpha 1

**CERS5**: Ceramide Synthase 5

**DGAT2**: Diacylglycerol O-Acyltransferase 2

**ATP6V1G2**: ATPase H+ Transporting V1 Subunit G2

**PLA2G4E**: Phospholipase A2 Group IVE

**ACSL1**: Acyl-CoA Synthetase Long Chain Family Member 1

**GPT2**: Glutamate Pyruvate Transaminase 2

**ALG11**: ALG11 Alpha-1,2-Mannosyltransferase

**KMO**: Kynurenine 3-Monooxygenase

**GANC**: Glucosidase Alpha, Neutral C

**B3GNT6**: UDP-GlcNAc:BetaGal Beta-1,3-N-Acetylglucosaminyltransferase 6

**LOC615045**: Uncharacterized LOC615045 (locus identifier)

**DPYD**: Dihydropyrimidine Dehydrogenase

**DGKI**: Diacylglycerol Kinase Iota

**PAFAH1B1**: Platelet Activating Factor Acetylhydrolase 1b Regulatory Subunit 1

**PFKFB4**: 6-Phosphofructo-2-Kinase/Fructose-2,6-Biphosphatase 4

**PFKFB3**: 6-Phosphofructo-2-Kinase/Fructose-2,6-Biphosphatase 3

**ASAH2**: N-Acylsphingosine Amidohydrolase 2

**MTMR3**: Myotubularin Related Protein 3

**MVK**: Mevalonate Kinase

**GMPS**: Guanine Monophosphate Synthase

**SGMS2**: Sphingomyelin Synthase 2

**PLD1**: Phospholipase D1

**PLD3**: Phospholipase D3

**PLD2**: Phospholipase D2

**GANAB**: Glucosidase II Alpha Subunit

**INPP5A**: Inositol Polyphosphate-5-Phosphatase A

**MAN2A2**: Mannosidase Alpha Class 2A Member 2

**MAN2A1**: Mannosidase Alpha Class 2A Member 1

**ST3GAL6**: ST3 Beta-Galactoside Alpha-2,3-Sialyltransferase 6

**XDH**: Xanthine Dehydrogenase

**NADK**: NAD Kinase

**FKTN**: Fukutin

**PLA2G2F**: Phospholipase A2 Group IIF

**AGK**: Acylglycerol Kinase

**GATB**: Glutamyl-tRNA Amidotransferase Subunit B

**FMO5**: Flavin Containing Dimethylaniline Monoxygenase 5

**NUDT9**: Nudix Hydrolase 9

**DHRS9**: Dehydrogenase/Reductase 9

**GLB1**: Galactosidase Beta 1

**ACTR3**: Actin Related Protein 3

**SYK**: Spleen Associated Tyrosine Kinase

**PLA2G4E**: Phospholipase A2 Group IVE

**INPPL1**: Inositol Polyphosphate Phosphatase Like 1

**RAF1**: Raf-1 Proto-Oncogene, Serine/Threonine Kinase

**PLD1**: Phospholipase D1

**WASF2**: WASP Family Member 2

**PLD2**: Phospholipase D2

**ACTR3**: Actin Related Protein 3

**ARFGEF2**: ADP Ribosylation Factor Guanine Nucleotide Exchange Factor 2

**GIT2**: GIT ArfGAP 2

**AGAP2**: ArfGAP With GTPase Domain, Ankyrin Repeat And PH Domain 2

**VPS26A**: VPS26 Retromer Complex Subunit A

**CBL**: Cbl Proto-Oncogene

**PLD1**: Phospholipase D1

**PLD2**: Phospholipase D2

**RAB31**: RAB31, Member RAS Oncogene Family

**PSD2**: Pleckstrin And Sec7 Domain Containing 2

**CXCR1**: C-X-C Motif Chemokine Receptor 1

**VPS45**: VPS45 Subunit of GARP Complex

**FGFR3**: Fibroblast Growth Factor Receptor 3

**RAB11FIP4**: RAB11 Family Interacting Protein 4

**SHC3**: SHC Adaptor Protein 3

**GNB4**: G Protein Subunit Beta 4

**CHMP1A**: Charged Multivesicular Body Protein 1A

**VPS36**: VPS36 Subunit of ESCRT Complex

**FGFR2**: Fibroblast Growth Factor Receptor 2

**INPP4A**: Inositol Polyphosphate-4-Phosphatase Type I A

**MTMR2**: Myotubularin Related Protein 2

**ITPR1**: Inositol 1,4,5-Trisphosphate Receptor Type 1

**PLCG2**: Phospholipase C Gamma 2

**HEYL**: Hes Related Family BHLH Transcription Factor With YRPW Motif Like

**EGLN2**: Egl-9 Family Hypoxia Inducible Factor 2

**COL4A2**: Collagen Type IV Alpha 2 Chain

**TXNRD1**: Thioredoxin Reductase 1

**WNT9A**: Wnt Family Member 9A

**IL12RB1**: Interleukin 12 Receptor Subunit Beta 1

**RELA**: RELA Proto-Oncogene, NF-KB Subunit

**KSR1**: Kinase Suppressor of Ras 1

**ITPR1**: Inositol 1,4,5-Trisphosphate Receptor Type 1

**RELA**: RELA Proto-Oncogene, NF-KB Subunit

**EFNA3**: Ephrin A3

**KSR1**: Kinase Suppressor of Ras 1

**RASGRF2**: Ras Protein Specific Guanine Nucleotide Releasing Factor 2

**KMT2D**: Lysine Methyltransferase 2D

**MTMR2**: Myotubularin Related Protein 2

**GALNT15**: Polypeptide N-Acetylgalactosaminyltransferase 15

**PGAP4**: Post-GPI Attachment to Proteins 4

**POMT1**: Protein O-Mannosyltransferase 1

**NAGA**: Alpha-N-Acetylgalactosaminidase

**GATB**: Glutamyl-tRNA Amidotransferase Subunit B

**GNS**: Glucosamine (N-Acetyl)-6-Sulfatase

**ENO4**: Enolase 4

**PLD3**: Phospholipase D3

**NUDT9**: Nudix Hydrolase 9

**INPP4A**: Inositol Polyphosphate-4-Phosphatase Type I A

**ADI1**: Acireductone Dioxygenase 1

**SARDH**: Sarcosine Dehydrogenase

**ACSS1**: Acyl-CoA Synthetase Short Chain Family Member 1

**EARS2**: Glutamyl-tRNA Synthetase 2, Mitochondrial

**AASS**: Aminoadipate-Semialdehyde Synthase

**PAFAH1B1**: Platelet Activating Factor Acetylhydrolase 1b Regulatory Subunit 1

**ITPR1**: Inositol 1,4,5-Trisphosphate Receptor Type 1

**TACR2**: Tachykinin Receptor 2

**ATP2A2**: ATPase Sarcoplasmic/Endoplasmic Reticulum Ca2+ Transporting 2

**ATP2B1**: ATPase Plasma Membrane Ca2+ Transporting 1

**ACTR3**: Actin Related Protein 3

**APC**: APC Regulator of WNT Signaling Pathway

**ARPC5L**: Actin Related Protein 2/3 Complex Subunit 5 Like

**ITGAE**: Integrin Subunit Alpha E

**FGF1**: Fibroblast Growth Factor 1

**APC**: APC Regulator of WNT Signaling Pathway

**E2F1**: E2F Transcription Factor 1

**BAK1**: BCL2 Antagonist/Killer 1

**FGF1**: Fibroblast Growth Factor 1

**PCYT1B**: Phosphate Cytidylyltransferase 1, Choline, Beta

**SELENOI**: Selenoprotein I

**PSD**: Pleckstrin And Sec7 Domain Containing

**GIT2**: GIT ArfGAP 2

**PDGFRA**: Platelet Derived Growth Factor Receptor Alpha

**WWP1**: WW Domain Containing E3 Ubiquitin Protein Ligase 1

**TGFBR2**: Transforming Growth Factor Beta Receptor 2

**PDGFRA**: Platelet Derived Growth Factor Receptor Alpha

**PDHA1**: Pyruvate Dehydrogenase E1 Subunit Alpha 1

**ERBB2**: Erb-B2 Receptor Tyrosine Kinase 2

**CDK6**: Cyclin Dependent Kinase 6

**ERBB2**: Erb-B2 Receptor Tyrosine Kinase 2

**TGFBR2**: Transforming Growth Factor Beta Receptor 2

**AGPAT5**: 1-acylglycerol-3-phosphate O-acyltransferase 5

**PTGFR**: Prostaglandin F receptor

**ARF2**: ADP-ribosylation factor 2

**SYK**: Spleen associated tyrosine kinase

**SPHK1**: Sphingosine kinase 1

**F2R**: Coagulation factor II thrombin receptor

**TSC1**: TSC complex subunit 1

**DGKZ**: Diacylglycerol kinase zeta

**GRM1**: Glutamate metabotropic receptor 1

**DNM1**: Dynamin 1

**PIK3R5**: Phosphoinositide-3-kinase regulatory subunit 5

**CYTH3**: Cytohesin 3

**MRAS**: Muscle RAS oncogene homolog

**GRM6**: Glutamate metabotropic receptor 6

**AKT2**: AKT serine/threonine kinase 2

**GNAS**: GNAS complex locus

**PLCB1**: Phospholipase C beta 1

**DGKI**: Diacylglycerol kinase iota

**ARF6**: ADP-ribosylation factor 6

AGPAT5 : 1-acylglycerol-3-phosphate O-acyltransferase 5

PLA2G2D: Phospholipase A2 group IID (note: sometimes mismatch with your PLA2G2D1; official is PLA2G2D)

PLA2G12A – Phospholipase A2 group XIIA

PCYT1A: Phosphate cytidylyltransferase 1A, choline

SAMD8: Sterile alpha motif domain containing 8

CHAT: Choline O-acetyltransferase

MBOAT1: Membrane bound O-acyltransferase domain containing 1

LPCAT1; Lysophosphatidylcholine acyltransferase 1

PLA2G3: Phospholipase A2 group III

DGKZ: Diacylglycerol kinase zeta

LCLAT1: Lysocardiolipin acyltransferase 1

PHOSPHO1: Phosphatase, orphan 1

DGKI: Diacylglycerol kinase iota

CASP9: Caspase 9

GADD45B : Growth arrest and DNA damage inducible beta

SESN3 : Sestrin 3

APAF1 : Apoptotic peptidase activating factor 1

CCNG2: Cyclin G2

PERP: P53 apoptosis effector related to PMP22

SESN1: Sestrin 1

PMAIP1: Phorbol-12-myristate-13-acetate-induced protein 1

THBS1: Thrombospondin 1

SERPINB5: Serpin family B member 5

TP73: Tumor protein p73

SMAD4: SMAD family member 4

PRKAB2: Protein kinase AMP-activated non-catalytic subunit beta 2

CDKN1B: Cyclin dependent kinase inhibitor 1B

SMAD3: SMAD family member 3

GADD45B: Growth arrest and DNA damage inducible beta

TGFB3 : Transforming growth factor beta 3

PLK2 : Polo like kinase 2

NLK: Nemo like kinase

KLF2: Kruppel like factor 2

GRM1: Glutamate metabotropic receptor 1

PRKAG3: Protein kinase AMP-activated non-catalytic subunit gamma 3

AKT2: AKT serine/threonine kinase 2

CCNG2: Cyclin G2

CAT: Catalase

S1PR1: Sphingosine-1-phosphate receptor 1

SKP2 : S-phase kinase associated protein 2

TLE4 : TLE family member 4, transcriptional corepressor

SMAD4: SMAD family member 4

WNT10A: Wnt family member 10A

FZD2: Frizzled class receptor 2

SMAD3: SMAD family member 3

WNT3A: Wnt family member 3A

CAMK2A: Calcium/calmodulin dependent protein kinase II alpha

PRICKLE2: Prickle planar cell polarity protein 2

PSEN1: Presenilin 1

NLK: Nemo like kinase

WNT16: Wnt family member 16

VANGL2 : VANGL planar cell polarity protein 2

APC: APC regulator of WNT signaling pathway

DAAM2: Dishevelled associated activator of morphogenesis 2

RSPO2: R-spondin 2

ROR1: Receptor tyrosine kinase like orphan receptor 1

PLCB1: Phospholipase C beta 1

ARF2: ADP-ribosylation factor 2

SMAD3: SMAD family member 3

IQSEC2: IQ motif and Sec7 domain ArfGEF 2

AP2A1: Adaptor related protein complex 2 subunit alpha 1

ARFGAP3: ADP-ribosylation factor GTPase activating protein 3

DNM1: Dynamin 1

CDC42: Cell division cycle 42

CYTH3: Cytohesin 3

EHD2: EH domain containing 2

RABEP1: Rabaptin, RAB GTPase binding effector protein 1

RAB31 : RAB31, member RAS oncogene family

PSD2 : Pleckstrin and Sec7 domain containing 2

KIF5C: Kinesin family member 5C

PIP5KL1: Phosphatidylinositol-4-phosphate 5-kinase like 1

RAB35: RAB35, member RAS oncogene family

CHMP4B: Charged multivesicular body protein 4B

LDLR: Low density lipoprotein receptor

SH3GL2: SH3 domain containing GRB2 like 2, endophilin A1

ARF5 : ADP-ribosylation factor 5

RAB11FIP4: RAB11 family interacting protein 4

ARF6: ADP-ribosylation factor 6

RYR2: Ryanodine receptor 2

ADCYAP1R1: ADCYAP receptor type I

PTCH1: Patched 1

F2R: Coagulation factor II thrombin receptor

CAMK2A: Calcium/calmodulin dependent protein kinase II alpha

ATP2B4: ATPase plasma membrane Ca2+ transporting 4

ATP2B3: ATPase plasma membrane Ca2+ transporting 3

ATP1B4: ATPase Na+/K+ transporting subunit beta 4

ATP2A2: ATPase sarcoplasmic/endoplasmic reticulum Ca2+ transporting 2

ATP2B2: ATPase plasma membrane Ca2+ transporting 2

CRHR2: Corticotropin releasing hormone receptor 2

VAV2: Vav guanine nucleotide exchange factor 2

NFKBIA: NFKB inhibitor alpha

LIPE: Lipase E, hormone sensitive type

AKT2: AKT serine/threonine kinase 2

ADORA1: Adenosine A1 receptor

MYL9: Myosin light chain 9

BVES: Blood vessel epicardial substance

CREB5: CAMP responsive element binding protein 5

PFKFB2: 6-phosphofructo-2-kinase/fructose-2,6-biphosphatase 2

LIPE: Lipase E, hormone sensitive type

PRKAB2: Protein kinase AMP-activated non-catalytic subunit beta 2

EEF2K: Eukaryotic elongation factor 2 kinase

SCD: Stearoyl-CoA desaturase

LEP: Leptin

TSC1: TSC complex subunit 1

EEF2: Eukaryotic elongation factor 2

ADRA1A: Adrenoceptor alpha 1A

CREB5: CAMP responsive element binding protein 5

DUSP2 : Dual specificity phosphatase 2

GADD45B: Growth arrest and DNA damage inducible beta

TGFB3: Transforming growth factor beta 3

CACNA2D2: Calcium voltage-gated channel auxiliary subunit alpha2delta 2

NLK: Nemo like kinase

EFNA5: Ephrin A5

VEGFA: Vascular endothelial growth factor A

DUSP7: Dual specificity phosphatase 7

CDC42: Cell division cycle 42

MRAS: Muscle RAS oncogene homolog

MAPK: MAPK activated protein kinase 3

TAOK3: TAO kinase 3

AKT2: AKT serine/threonine kinase 2

RAPGEF2: Rap guanine nucleotide exchange factor 2

FLNA: Filamin A

MAP3K9: Mitogen-activated protein kinase kinase kinase 9

PTPN5: Protein tyrosine phosphatase non-receptor type 5

MAP4K4: Mitogen-activated protein kinase kinase kinase kinase 4

NLGN3: Neuroligin 3

SDC3: Syndecan 3

F11R: F11 receptor

SELE: Selectin E

VCAN: Versican

MPZ: Myelin protein zero

CLDN12: Claudin 12

CD58: CD58 molecule

TIGIT: T cell immunoreceptor with Ig and ITIM domains

NECTIN3: Nectin cell adhesion molecule 3

CD34: CD34 molecule

ICOSLG: Inducible T cell costimulator ligand

PTGIR: Prostaglandin I2 receptor

SYK: Spleen associated tyrosine kinase

AKT2: AKT serine/threonine kinase 2

ITGB3: Integrin subunit beta 3

SNAP23: Synaptosome associated protein 23

F2R: Coagulation factor II thrombin receptor

GNAS: GNAS complex locus

BTK: Bruton tyrosine kinase

TLN2: Talin 2

PLCB1: Phospholipase C beta 1

PIK3R5: Phosphoinositide-3-kinase regulatory subunit 5

CDKN1B: Cyclin dependent kinase inhibitor 1B

PTGER1: Prostaglandin E receptor 1

CAMK2A: Calcium/calmodulin dependent protein kinase II alpha

ETS1: ETS proto-oncogene 1, transcription factor

CDC42: Cell division cycle 42

CASP9: Caspase 9

AKT2: AKT serine/threonine kinase 2

E2F1: E2F transcription factor 1

PMAIP1: Phorbol-12-myristate-13-acetate-induced protein 1

E2F3: E2F transcription factor 3

SKP2: S-phase kinase associated protein 2

JAK3: Janus kinase 3

IL13RA1: Interleukin 13 receptor subunit alpha 1

NKX3-1: NK3 homeobox 1

ARNT2: Aryl hydrocarbon receptor nuclear translocator 2

SMAD4: SMAD family member 4

WNT10A: Wnt family member 10A

FZD2: Frizzled class receptor 2

SMAD3: SMAD family member 3

IL4R: Interleukin 4 receptor

GADD45B: Growth arrest and DNA damage inducible beta

NOS2: Nitric oxide synthase 2

APAF1: Apoptotic peptidase activating factor 1

TGFB3: Transforming growth factor beta 3

WNT3A: Wnt family member 3A

TXNRD1: Thioredoxin reductase 1

PTCH1: Patched 1

STAT2: Signal transducer and activator of transcription 2

F2R: Coagulation factor II thrombin receptor

WNT16: Wnt family member 16

VEGFA: Vascular endothelial growth factor A

NFKBIA: NFKB inhibitor alpha

APC: APC regulator of WNT signaling pathway

GNAS: GNAS complex locus

PLCB1: Phospholipase C beta 1
